# No Change in Illicit Tobacco Use Following the Introduction of Standardised Packaging? A Longitudinal Online Survey in the United Kingdom

**DOI:** 10.1177/1179173X251405166

**Published:** 2025-12-18

**Authors:** Daniel Jones, Catherine Best, Crawford Moodie

**Affiliations:** 1 Institute for Social Marketing and Health, Faculty of Health Sciences and Sport, University of Stirling, Stirling, UK; 2 Local Health and Global Profits, Department for Health, University of Bath, Claverton Down, Bath, UK

**Keywords:** standardised packaging, illicit tobacco, tobacco control policy, longitudinal, survey

## Abstract

**Background:**

The tobacco industry has argued for decades that standardised packaging would lead to an increase in illicit tobacco use, but this is not supported by current evidence.

**Objective:**

To explore longer-term associations between standardised packaging and illicit tobacco use.

**Design:**

The Adult Tobacco Policy Survey is a longitudinal online survey with people who smoke or who previously smoked aged 16 and older in the United Kingdom (UK), with one wave pre-standardised packaging (2016) and three waves post-standardised packaging (2017, 2019, 2022).

**Methods:**

Participants who smoke and who bought cigarettes or roll-your-own (RYO) tobacco were asked where they last bought, and usually buy, cigarettes or RYO tobacco to identify illicit purchasing channels. They were also asked whether they purchased potentially illicit cigarettes or RYO (with no or incorrect Warnings, Smuggled, or Fake (WSF) in the last three months and, if so, how often, why, and price paid.

**Results:**

People who smoke were less likely to report buying illicit (WSF) cigarettes or RYO in 2019 (11.2%) and 2022 (11.4%) relative to 2016 (13.2%), although this only remained significant in adjusted models in 2022. For participants that purchased or received any illicit (WSF) tobacco product, low cost, high availability and opportunism were the key reasons for doing so. In comparison, relatively few people who smoke reported ‘packs looking nice’ as a reason for purchasing illicit tobacco pre- and post-standardised packaging.

**Conclusion:**

Evidence from four waves of longitudinal data suggests that there was no increase in illicit tobacco use in the UK following the introduction of standardised packaging.

## Introduction

Illicit tobacco is tobacco sold without duty paid and which may not comply with other regulatory requirements (e.g. minimum pack size) or safety requirements (e.g. use of self-extinguishing paper).^1,2^ It includes genuine products smuggled from abroad (i.e. illegally moved into the country), counterfeit (fake) products that resemble genuine brands, unbranded tobacco, and ‘cheap whites’ (legitimately manufactured brands intentionally sold on the illegal market).^1,2^ It is a significant public health issue globally given its low price, as well as high availability and accessibility, may increase use and amount consumed and reduce motivation to quit.^3-9^ Illicit tobacco is often incorrectly labelled (e.g. displaying no or the wrong health warnings) and/or has inferior quality packaging, is sold ‘under the counter’ (e.g. in markets or non-retail venues) and to underage people, and is associated with tax evasion, organised crime and smuggling of other commodities (e.g. weapons, drugs).^2,4,5,10^

Evidence from 84 countries indicates that 11.6% of cigarette consumption globally in 2007 was illicit,^11^ while a literature review across 36 countries suggested that, on average, illicit cigarettes accounted for 11.2% of the market from 2010 to 2018 and that eliminating illicit cigarettes would decrease cigarette consumption by 1.9% across these countries.^12^ Notwithstanding geographical and methodological differences, that these studies found similar estimates suggests a relative stability in the proportion of global illicit trade in a shrinking cigarette market.^13^ Europe has the highest number of seized cigarettes globally,^5^ with the European Anti-Fraud Office (OLAF) reporting that 616 million illicit cigarettes, 140 tonnes of raw tobacco and 6 tonnes of water pipe tobacco were seized in 2023.^14^ The World Health Organization estimates that Europe would gain €9-11 billion in revenue by eliminating illicit tobacco.^5^ Despite reported overall decreases of illicit tobacco in the UK, which has decreased from 21.7% in 2005/2006 to 16.6% in 2019/2020 for both cigarettes and roll-your-own (RYO), according to HM Revenue and Customs it cost the government an estimated £2.5 billion in lost revenue (VAT and excise duty) in 2021 to 2022.^15^

Standardised (‘plain’) tobacco packaging broadly refers to the requirement for tobacco products to be sold in packs that are the same base colour and that are devoid of any branding except brand variant name, which must be in a standardised font type and size. At least 20 countries/territories having implemented the measure.^16^ Since May 2017, following a 12-month sell-through period, cigarettes and RYO in the UK must be sold in (drab dark brown) standardised packs.^17,18^ The legislation also requires pictorial warnings to cover at least 65% of the primary pack display areas and two additional text messages (‘Smoking kills – quit now’, and ‘Tobacco smoke contains over 70 substances known to cause cancer’) on the secondary pack display areas (i.e. the lateral surfaces of cigarette packs and, while this varies by pack type, the pocket area and inner flap of RYO pouches).^17,18^ Additionally, cigarettes must be sold in packs with a minimum of 20 cigarettes and RYO in packs with a minimum of 30 grams.^17,18^ Tobacco companies have opposed standardised packaging for decades,^19,20^ forecasting an increase in illicit tobacco.^1,2^ They argue that the policy would make it easier to counterfeit packs and therefore harder for customers to distinguish between legitimate and counterfeit products, and may also increase the appeal of fully-branded illicit packs.^1,2,10,13,21-24^ However, evidence suggests neither availability nor use of illicit tobacco has been significantly affected by standardised packaging,^1,22,23,25,26^ with a time-series analysis finding no increase in illicit tobacco and cross-border purchases in England post-implementation.^9^

The impacts of illicit tobacco require continued attention from the public health community because it offers a cheap alternative to licit tobacco and undermines tobacco control policy.^27^ As most studies exploring the association between illicit tobacco and standardised packaging rely on pre-implementation or short-term data,^9,22,23,25^ research examining longer-term associations is needed,^21^ not least as this may help to support policy adoption elsewhere.^28^ We explored whether people who smoke in the UK had purchased cigarettes or RYO that may have been illicit in the last three months as well as reason for purchase, last and usual source of purchase, and price paid.

## Methods

### Design and Sample

The Adult Tobacco Policy Survey (ATPS) is a longitudinal online survey in the UK with people who smoke or who previously smoked. The sample was recruited from the online panel of market research company YouGov, which includes over 800 000 people aged 16 and over in the UK.^29^ Panel members are invited to participate in surveys and receive an incentive (in the form of points that can be redeemed for shopping vouchers) for doing so. For the ATPS, email invitations with a survey link were sent by YouGov to panel members whose profiling data suggested they were people who smoke. For those who clicked on the link they were asked a screening question about their smoking status. The inclusion criterion at Wave (W) 1 was that participants reported smoking cigarettes or RYO within the last three months. Those indicating that they smoked within the last three months were provided with an information and consent page at the start of the survey (see Supplementary file for questionnaire). Exclusion criterion, at each wave, was lack of informed consent to participate in the survey.

Recruitment for Wave (W) 1, conducted pre-standardised packaging (April-May 2016), was by quota sampling for age, gender, government office region, and tobacco consumption to represent the national profile of people who smoke aged 16 and over in the UK, based on the Opinions and Lifestyle Survey and Integrated Household Survey.^30^ The target sample at W1 was 6000 people who smoke, with this driven by practical (cost) considerations. The survey was piloted with 100 participants (2% of the W1 sample) at each wave. At W1, 6233 participants who reported smoking cigarettes within the past three months were recruited. All W1 participants were eligible for inclusion at subsequent waves. Of those recruited at W1, 4293 (3629 were people who smoked, 607 previously smoked, 36 did not smoke cigarettes or RYO but smoked another form of tobacco, 7 reported no smoking for the past 3 months, 14 missing data) responded at W2 (September-November 2017), 3175 (2412 were people who smoked, 700 previously smoked, 44 did not smoke cigarettes or RYO but smoked another form of tobacco, 6 reported no smoking for the past 3 months, 13 missing data) at W3 (May-July 2019), and 3047 (1935 were people who smoked, 1052 previously smoked, 45 did not smoke cigarettes or RYO but smoked another form of tobacco, and 15 did not know their smoking status) at W4 (October-November 2022).

Sample characteristics across the four waves are provided in Supplemental Table 1, with the subsample who reported buying illicit tobacco products (no/incorrect Warnings, Smuggled or Fake [WSF]) and those who did not shown in Table 1. The study received ethical approval from the General University Ethical Panel (GUEP 8359) at the University of Stirling. At W1, the ethics panel waived the need for parental consent for 16-17-year-olds in line with the guidance from the UK’s Health Research Authority.^31^

**Table 1.** Characteristics of Subsample Who Never or Ever Buy Illicit Tobacco Products

Variable	Categories	Reported illicit purchase at one wave or more		
		Never bought illicit	Ever bought illicit	Total
		N (%)	N (%)	N (%)
Age at Wave 1	16-24	469 (75.7)	151 (24.3)	620 (100)
	25-39	1367 (77.6)	395 (22.4)	1762 (100)
	40-55	1610 (79.2)	422 (20.8)	2032 (100)
	56+	1422 (82.8)	295 (17.2)	1717 (100)
Sex	Male	2185 (76.6)	669 (23.4)	2854 (100)
	Female	2683 (81.9)	594 (18.1)	3277 (100)
Education level at Wave 1	High school or less	1519 (75.7)	487 (24.3)	2006 (100)
	Technical, trade school, A levels, or community college	1264 (79.9)	318 (20.1)	1582 (100)
	At least university degree	1944 (82.4)	416 (17.6)	2360 (100)
	Don’t know or prefer not to say	141 (77.1)	42 (22.9)	183 (100)
Gross household income at Wave 1	Under £30 000	2149 (76.8)	651 (23.3)	2800 (100)
	£30 000 to £44 999	1027 (80.1)	255 (19.9)	1282 (100)
	£45 000 and over	775 (81.3)	178 (18.7)	953 (100)
	Don’t know or prefer not to answer	917 (83.7)	179 (16.3)	1096 (100)
Ethnic group	White British	4345 (80.2)	1075 (19.8)	5420 (100)
	White other	253 (73.8)	90 (26.2)	343 (100)
	Other ethnic group	222 (71.6)	88 (28.4)	310 (100)
	Prefer not to say	48 (82.8)	10 (17.2)	58 (100)
Occupational social grade	ABC1	2867 (81.4)	654 (18.6)	3521 (100)
	C2DE	1870 (76.8)	564 (23.2)	2434 (100)
	Not known/prefer not to say	131 (74.4)	45 (25.6)	176 (100)
Heaviness of smoking index at Wave 1	Low	2164 (83.0)	444 (17.0)	2608 (100)
	Medium	2434 (77.4)	711 (22.6)	3145 (100)
	High	203 (69.1)	91 (30.9)	294 (100)
	Missing	67 (79.8)	17 (20.2)	84 (100)
	Total	4868 (79.4)	1263 (20.6)	6131 (100)

Occupational social grade: ABC1 signifies more advantaged groups; C2DE signifies working-class groups. We defined ‘illicit’ tobacco products as those with no/incorrect warnings, smuggled or fake (WSF).

### Measures

#### Demographics and Smoking Behaviour

Information was gathered regarding age, sex, occupational social grade (ABC1, signifying more advantaged groups; C2DE, signifying less advantaged groups),^32^ household income, education, ethnic group, smoking status at the time of the survey, and heaviness of smoking index (HSI).^33^ Table 1 shows participants’ demographic and smoking behaviours, split by those reporting obtaining illicit (WSF) tobacco at any wave vs those who did not.

#### Frequency of Purchase of Illicit Tobacco Products

Participants who reported buying cigarettes or RYO were asked, in a grid response format, ‘In the last three months how many times, if at all, have you bought/got packs of cigarettes or rolling tobacco in the UK that…? A: Did not have health warnings, or had health warnings in a language other than English? B: Might have been smuggled? C: Might be fake (i.e., copies of real brands)? The authors developed this question to explore different ways in which participants may be able to identify whether their tobacco purchases may be illicit. For each question the response options were ‘Never’, ‘Once’, ‘2-5 times’, ‘6-10 times’, ‘More than 10 times’, and ‘Don’t know’. Participants responding ‘Once’, ‘2-5 times’, ‘6-10 times’ or ‘More than 10 times’ to any question were coded as having used illicit tobacco at that wave. For analysis, responses were recoded as ‘Never’, ‘1-5 times’, ‘More than 5 times’ and ‘Don’t know’. This, and the all the illicit questions, remained the same across the four survey waves.

#### Reasons for Illicit Purchase

For packs which may have been illicit (WSF), participants were asked why they bought them (‘They were cheap’, ‘I got them while travelling outside the UK’, ‘They were available in my local area’, ‘A friend, family member or work colleague sometimes has them’, ‘The packs looked nice’, ‘Other’, and ‘Don’t know’). Participants could select more than one option. This question was developed by the authors based on factors associated with illicit tobacco use.^6^

#### Price Paid for Illicit Tobacco Products

For packs which may have been illicit (WSF), participants were asked how much they normally pay for them. Response options were: ‘Same price as I would pay at a shop for cigarettes or RYO (‘regular price’)’, ‘Less than 10% cheaper than regular price’, ‘11-20% cheaper than regular price’, ‘21-30% cheaper than regular price’, ‘31-40% cheaper than regular price’, ‘41-50% cheaper than regular price’, ‘51-60% cheaper than regular price’, ‘61-70% cheaper than regular price’, ‘More than 70% cheaper than regular price’ and ‘Don’t know’. This question was created by the team to provide more granular detail on cost paid.

#### Source of Purchase: Last and Usual

Aside from directly asking whether participants believed that they had bought illicit cigarettes (e.g. that were smuggled or fake), we also assessed potential illicit purchase by asking where participants last bought cigarettes or RYO and where they usually buy them, similar to past research.^9^ The response options were split into *licit* sources (‘Supermarket’, ‘Newsagent \ Off licence \ Corner shop’, ‘Petrol station’, and ‘Duty-free shop’); *potentially illicit* sources (‘Outside the UK, but NOT a duty-free shop’, ‘From friends, relatives or work colleagues’, ‘Pub (behind the bar)’ and ‘Internet’); and *illicit* sources (‘Newsagent \ Off licence \ Corner shop (‘cheaply, under the counter’)’, ‘Pub (someone who comes round selling cigarettes/RYO cheaply)’, ‘Someone who sells cigarettes/RYO cheaply on the street or from a house or flat’, ‘Someone who sells cigarettes/RYO cheaply at a market stall, car boot sale or from a mobile van’), with ‘Other’, ‘Have not bought’, and ‘Don’t know’ options.

### Analysis

Differences in participant characteristics between those purchasing illicit (WSF) tobacco vs those who did not purchase illicit (WSF) at any time point were tested with Fisher’s exact test. Using generalised estimating equations (GEE) with an exchangeable working correlation structure, we examined change in the proportion that gave a response other than ‘Never’ or ‘Don’t know’ to at least one type of illicit (WSF) tobacco across all survey waves. Results are presented as the adjusted odds ratio (aOR) and its 95% confidence interval.

Whether the difference in probability of obtaining any illicit (WSF) tobacco between occupational social grades changed across survey waves was tested by including an interaction term in the model. The marginal proportions and their 95% confidence intervals are presented graphically. Analyses included only those who smoked in the last 3 months and had purchased cigarettes or tobacco. A sensitivity analysis was conducted restricting the sample to complete cases, i.e. those who had responded in all four survey waves.

The trajectory of potential illicit (WSF) tobacco purchases across all waves was examined graphically. The confidence intervals for the proportions purchasing from licit, potentially illicit, and illicit sources are derived from a mixed effects ordinal regression with a random effect of participant. All analyses were conducted in Stata version 17 and adjusted for sex, age group at baseline, education, gross household income, ethnic group and HSI at baseline. These covariates were chosen to adjust for changes to the cohort composition across survey waves due to smoking cessation. The threshold for statistical significance was set to 0.05.

## Results

### Sample Characteristics

Sample characteristics across the four survey waves are shown in Supplemental Table 1, with the subsample who reported buying illicit (WSF) tobacco products and those who did not shown in Table 1. Participants who reported buying illicit (WSF) tobacco products at one wave or more (*n* = 1263, 20.2%) were less likely to identify as White British (*n* = 1075, 85.1% vs *n* = 4345, 89.3% in never illicit) and more likely to be male (*n* = 669, 53.0% vs *n* = 2185, 44.9% in never illicit), with medium or high HSI (*n* = 802, 63.5% vs n = 2637, 54.2% in never illicit), high school education or less (*n* = 487, 38.6% vs *n* = 1519, 31.2% in never illicit), and under 40 years-old (*n* = 546, 43.2% vs *n* = 1836, 37.7% in never illicit) at W1. These differences were all statistically significant by Fisher’s exact test.

### Purchase of Illicit Tobacco Products

At W1 (2016), 13.2% of participants who smoke reported that they had bought illicit (WSF) cigarettes/tobacco in the last 3 months, with this figure 13.1% at W2 (2017), 11.4% at W3 (2019), and 11.2% at W4 (2022). The GEE analysis indicated that, relative to W1, participants were less likely to report buying illicit (WSF) cigarettes in W3 (unadjusted odds ratios (OR) = 0.87, 95% CI 0.77-0.99) and W4 (OR = 0.83, 95% CI 0.72-0.96). After adjustment for sociodemographic and smoking variables at baseline, the difference between W1 and W3 was no longer statistically significant (aOR = 0.89, 95% CI 0.78-1.02); the difference between W1 and W4 remained significant (aOR = 0.85, 95% CI 0.74-0.99). The difference between W1 and W2 was not significant in adjusted or unadjusted models (OR = 1.00, 95% CI 0.90-1.11, aOR = 1.04, 95% CI 0.93-1.15). A sensitivity analysis restricted to participants who responded at all four survey waves found the same pattern of results, see Supplemental Table 4.

### Purchase of Illicit Tobacco Products by Occupational Social Grade

**Figure 1.**
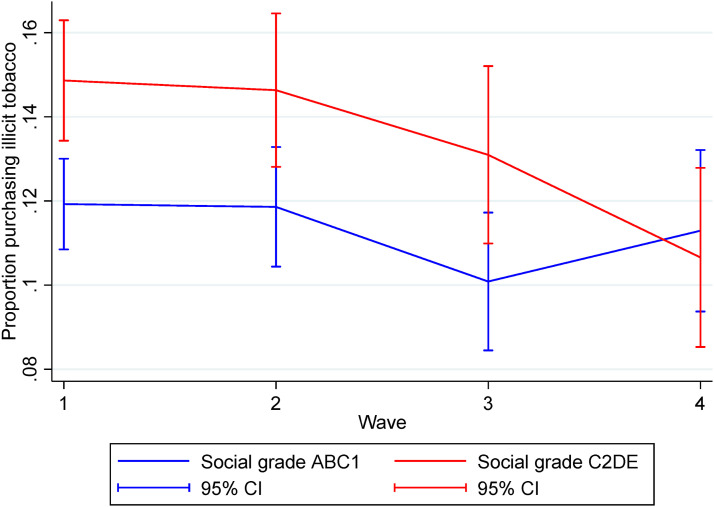
Proportion of participants reporting purchase of illicit tobacco by occupational social grade and survey wave The graph shows the marginal predicted proportions from a generalised estimating equation with binomial family, logit link, and exchangeable correlation structure. Occupational social grade (ABC1 signifies more advantaged groups; C2DE signifies less advantaged groups) and wave are included as an interaction and the model is adjusted for sex, age group at baseline, education, gross household income, ethnic group and HSI at baseline. We defined ‘illicit’ tobacco products as those with no/incorrect warnings, smuggled or fake (WSF).

Figure 1 shows the proportion of participants reporting purchase of illicit (WSF) tobacco by occupational social grade. The difference between occupational social grades in the proportion purchasing illicit (WSF) tobacco was significant overall, with participants in occupational social grade C2DE more likely to purchase than those in ABC1 (OR = 1.28, 95% CI 1.10-1.50). However, groups did not differ in the magnitude of their change over time as determined by the interaction effect between occupational social grade and survey wave (effect of occupational social grade C2DE vs ABC1 at W2 relative to W1, OR = 1.00, 95% CI 0.81-1.24; effect of occupational social grade C2DE vs ABC1 at W3 relative to W1, OR = 1.02, 95% CI 0.79-1.32; effect of occupational social grade C2DE vs ABC1 at W4 relative to W1, OR = 0.76, 95% CI 0.57-1.02). In a sensitivity analysis, restricted to participants who completed all four survey waves, the effect of occupational social grade at W4 vs W1 was statistically significant. This indicates that in the complete cases, people from occupational social grade C2DE are more likely than people in ABC1 to purchase illicit (WSF) at W1 but not at W4 (see Supplemental Table 4 and Supplemental Figure 1).

### Frequency of, and Reasons for, Illicit Purchase

Most participants reported having never purchased cigarettes or rolling tobacco that may have been illicit (WSF). Participants who bought potentially illicit (WSF) tobacco products at any wave typically did so 5 times or fewer and because they were cheap, available (e.g. local area, friends/family) and obtained outside the UK (opportunism), see Table 2. Reduced cost was the key reason for illicit (WSF) purchase across all waves. The look of packs, which is a key impact of standardised packaging, remained a relatively infrequent reason given for the purchase of illicit (WSF) tobacco across all survey waves. Among participants who gave ‘other’ as a reason, some stated in the free text section that they had purchased by mistake or given a friend money to buy them when abroad.

**Table 2.** Frequency and Reasons for Buying/Receiving Packs of Cigarettes or RYO in the UK That May Have Been Illicit

	Wave 1	Wave 2	Wave 3	Wave 4
Frequency of purchase of cigarettes/RYO with no or incorrect warnings	N (%)	N (%)	N (%)	N (%)
Never	5285 (86.3)	3134 (78.7)	2118 (88.7)	1717 (89.1)
1-5 times	405 (6.6)	222 (6.2)	126 (5.3)	109 (5.7)
More than 5 times	164 (2.7)	118 (3.3)	56 (2.3)	41 (2.1)
Don’t know	267 (4.4)	99 (2.8)	89 (3.7)	61 (3.2)
Reason for purchase of cigarettes/RYO with no or incorrect warnings	N (%)	N (%)	N (%)	N (%)
They were cheap	217 (38.1)	116 (34.1)	77 (42.3)	56 (37.3)
I got them outside the UK	104 (18.3)	84 (24.7)	56 (30.8)	36 (24.0)
They were available in my local area	136 (23.9)	66 (19.4)	38 (20.9)	27 (18.0)
A friend, family member or work colleague sometimes has them	158 (27.8)	81 (23.8)	43 (23.6)	33 (22.0)
The packs looked nice	32 (5.6)	24 (7.1)	15 (8.2)	16 (10.7)
Other	38 (6.7)	27 (7.9)	4 (2.2)	20 (13.3)
Don’t know	55 (9.7)	40 (11.8)	16 (8.8)	19 (12.7)
Frequency of purchase of cigarettes/RYO believed to be smuggled	N (%)	N (%)	N (%)	N (%)
Never	5254 (85.8)	3119 (87.3)	2073 (86.8)	1696 (88.0)
1-5 times	335 (5.5)	172 (4.8)	121 (5.1)	88 (4.6)
More than 5 times	107 (1.8)	69 (1.9)	36 (1.5)	24 (1.2)
Don’t know	425 (6.9)	213 (6.0)	159 (6.7)	120 (6.2)
Reason for purchase of cigarettes believed to be smuggled	N (%)	N (%)	N (%)	N (%)
They were cheap	256 (57.9)	124 (51.5)	91 (58.0)	67 (59.8)
I got them outside the UK	37 (8.4)	20 (8.3)	14 (8.9)	14 (12.5)
They were available in my local area	126 (28.5)	66 (27.4)	47 (29.9)	33 (29.5)
A friend, family member or work colleague sometimes has them	147 (33.3)	78 (32.4)	49 (31.2)	33 (29.5)
The packs looked nice	31 (7.0)	15 (6.2)	6 (3.8)	10 (8.9)
Other	10 (2.3)	11 (4.6)	2 (1.3)	6 (5.4)
Don’t know	14 (3.2)	6 (2.5)	4 (2.6)	7 (6.3)
Frequency of purchase of cigarettes/RYO believed to be fake	N (%)	N (%)	N (%)	N (%)
Never	5430 (88.7)	3207 (89.8)	2140 (89.6)	1733 (89.9)
1-5 times	275 (4.5)	140 (3.9)	89 (3.7)	71 (3.7)
More than 5 times	40 (0.7)	31 (0.9)	24 (1.0)	26 (1.4)
Don’t know	376 (6.1)	195 (5.5)	136 (5.7)	98 (5.1)
Reason for purchase of cigarettes believed to be fake	N (%)	N (%)	N (%)	N (%)
They were cheap	151 (47.9)	80 (46.8)	57 (50.4)	55 (56.7)
I got them outside the UK	29 (9.2)	21 (12.3)	10 (8.9)	10 (10.3)
They were available in my local area	74 (23.5)	46 (26.9)	33 (29.2)	21 (21.7)
A friend, family member or work colleague sometimes has them	84 (26.7)	39 (22.8)	28 (24.8)	16 (16.5)
The packs looked nice	29 (9.2)	17 (9.9)	7 (6.2)	8 (8.3)
Other	24 (7.6)	10 (5.9)	3 (2.7)	8 (8.3)
Don’t know	12 (3.8)	9 (5.3)	9 (8.0)	9 (9.3)

### Price Paid for Illicit Tobacco

The most common price paid for tobacco that may have been illicit (WSF) was 41-50% lower than the standard price of tobacco. That is, 17.3% (*n* = 26) of those who purchased cigarettes/RYO with no or incorrect warnings at W4 reported the price to be 41-50% cheaper than in a shop. Similar proportions of participants reported buying tobacco 41-50% cheaper when purchasing cigarettes believed to be smuggled (*n* = 25, 22.3%) or fake (*n* = 22, 22.7%) at W4.

### Source of Purchase: Last and Usual

**Figure 2.**
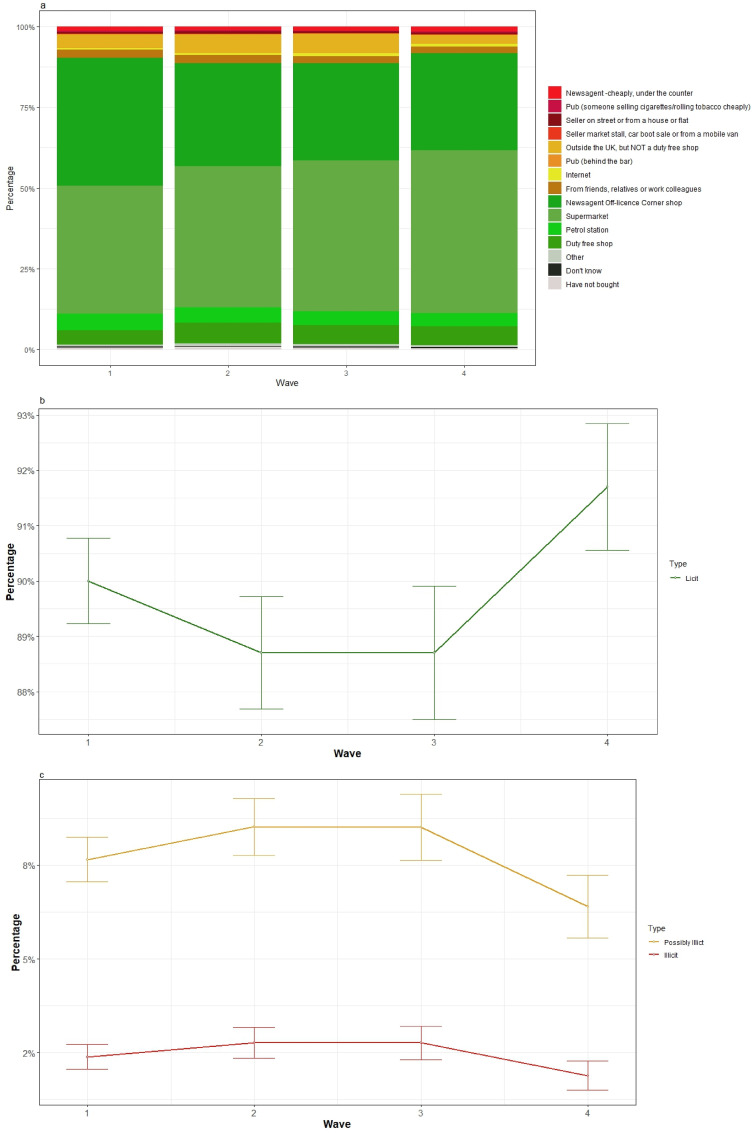
Source of last cigarette purchase by survey wave (A) shows the proportion of participants who reported each of these sources as where they last purchased cigarettes at wave 1 (April-May 2016), wave 2 (Sept-Nov 2017), wave 3 (May-July 2019) and wave 4 (Oct-Nov 2022). (B, C) show these proportion collapsed into licit, possibly illicit and illicit categories. The 95% confidence intervals for the proportions are shown at each wave and these are derived from a mixed effects ordinal model with a random effect of participant.

**Figure 3.**
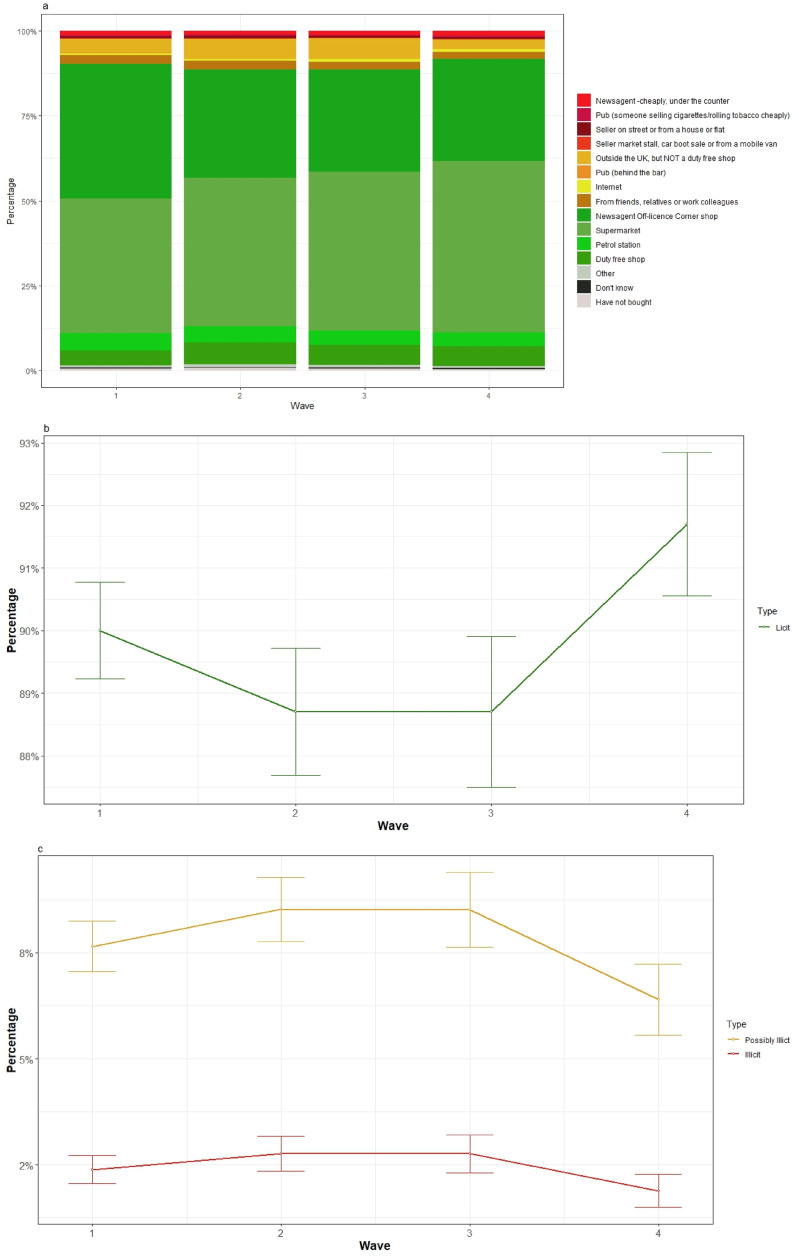
Source of usual purchase by survey wave (A) shows the proportion of participants who reported each of these sources as where they last pruchased cigarettes at wave 1 (April-May 2016), wave 2 (Sept-Nov 2017), wave 3 (May-July 2019) and wave 4 (Oct-Nov 2022). (B, C) show these prortions collapsed into illict, possibly illict amnd illicit categories. The 95% confidence intevrals for the proportions are shown at each wave and these are derived from a mixed effects ordinal model with a random effect of participant.

Figures 2 and 3 (and Supplemental Tables 2 and 3) provide the last and usual source of cigarette/RYO purchase, respectively. The proportions of participants reporting purchasing from each source by survey wave is presented, with this relatively stable. Across all waves, the most common sources were licit, particularly supermarkets and newsagents/off licences/corner shops. Potentially illicit sources were typically outside the UK but not a duty-free shop, and friends, relatives or work colleagues. The most common, albeit infrequent, illicit sources for both last and usual purchase were ‘cheaply, under the counter at newsagents/off licences/corner shops’ and from ‘someone who sells products cheaply on the street or from a house or flat’. Figures 2B and 3B show the proportions collapsed over the categories ‘licit’, ‘possibly illicit’ and ‘illicit’. The proportions in each of these key categories is shown over survey wave and with their 95% confidence intervals to give a sense of the uncertainty around these estimates, which can be seen to be low in this sample. The figures indicate that there is little change in the proportions across all four survey waves.

## Discussion

We conducted a longitudinal survey in the UK between 2016 and 2022, finding that most participants who smoke indicated that they did not engage with illicit (WSF) cigarettes or RYO. This is consistent with a wider decline in the UK illicit market.^15^ Those who reported their last or usual purchase to be illicit (WSF) cigarettes or RYO typically acquired this ‘cheaply, under the counter’ at newsagents, off licences or corner shops, or from someone who sells products cheaply on the street or from a house or flat. We found no evidence that compared to W1, self-reported illicit (WSF) cigarettes or RYO use was higher at W2, W3 of W4, corresponding with the introduction of standardised packaging.

Most participants reported low cost (often 41-50% cheaper than regular price), availability (e.g. local area, friends/family) and opportunism (while outside UK) as key reasons for purchasing and receiving potentially illicit (WSF) tobacco products, consistent with past research.^6,34-36^ A smaller number of participants reported purchasing illicit (WSF) cigarettes or RYO, including products with no or incorrect warnings, because the packs looked nice. A discrete choice experiment in Canada found that nearly half of participants preferred cheaper illicit cigarettes in a fully-branded pack without any pictorial warnings, regardless of the licit cigarette alternatives.^37^ Both studies suggest that fully-branded packaging with no or weaker warnings may be more appealing to some who smoke, but our real-world evaluation suggests that this is not the key driver of illicit purchase, and it is not associated with an increase in illicit tobacco use.

We found that participants from less advantaged groups (occupational social grade C2DE) were more likely to report purchasing illicit (WSF) tobacco products than those in more advantaged groups (ABC1). This reflects the situation in the UK, where illicit tobacco is disproportionately used by less advantaged groups,^4,36,38^ with use primarily driven by cost.^36^ To reach the UK Government’s ‘smokefree’ target (defined as less than 5% adult smoking prevalence) by 2030,^39^ there is a need to reach those in more deprived communities. Targeted support, such as community outreach to engage those who may not be able to access stop-smoking services,^40^ and addressing barriers to quitting, such as the availability of cheap illicit tobacco which may have no or incorrect health warnings, could help to promote long-term quit success and reduce future smoking-related inequalities among disadvantaged groups.^41^ While price policy and taxation are key tobacco control methods globally,^42,43^ with the greatest impact on young and disadvantaged people,^43,44^ strategies to reduce tobacco consumption and improve public health are being undermined by access to significantly cheaper tobacco products.

According to tobacco industry estimates,^45-47^ 80% of people who smoke in the UK purchased illicit tobacco products (not subject to UK tax) at least once in 2023. While we measured illicit (WSF) purchasing over a shorter period (last 3-6 months), and only until 2022, the discrepancy is stark. An evaluation of tobacco company submissions to the UK consultation on standardised packaging in 2012 concluded that industry-connected evidence was of very low quality, particularly compared to independent evidence, with tobacco companies relying on evidence they commissioned and the opinions of third-parties with financial links to the industry.^48^ It is argued that tobacco companies attempt to influence policies by increasing the perception that illicit trade is a significant and growing problem,^24,27,49,50^ which is inconsistent with historical trends and independent data.^51^ Indeed, global efforts to reduce illicit tobacco trade are accelerating, with progress achieved in several regions.^13^

Contrary to industry concerns, some research suggests that standardised packaging is associated with a reduction in illicit tobacco.^10^ A time series analysis in the UK found a negative relationship between the implementation of standardised packaging and illicit tobacco and cross-border purchases.^9^ Despite differences between the two studies, e.g. Vincent et al^9^ explored illicit tobacco via a repeat cross-sectional design, used slightly different questions (e.g. ‘under the counter’ vs ‘cheaply, under the counter’) and a different timeframe for illicit purchase (last 6 months vs last 3 months), the findings are comparable as we found that participants were less likely to report buying illicit (WSF) cigarettes or RYO in W3 (2019) and W4 (2022) relative to W1 (2016); after adjustment for sociodemographic and smoking variables at W1, only the difference between W1 and W4 remained significant.

Aside from changing the appearance of packs, in the UK standardised packaging regulations also set a minimum pack size of 20 for cigarettes and 30 grams for RYO. Concomitant with these larger pack sizes, the price of packs increased post-standardised.^51,52^ While the tobacco industry claims that higher prices are associated with increased use of illicit tobacco products,^53^ this is not supported by our data. Another key regulatory change occurred between W3 and W4, a ban on characterising flavours in cigarettes.^18^ That reported illicit (WSF) cigarette or RYO use was extremely similar in W3 and W4 suggests that the flavour ban did not affect illicit (WSF) use among our sample, at least short term. Studies in Canada, England and the Netherlands similarly found no evidence of an increase in illicit cigarette purchasing following flavour cigarette bans.^54-56^ This provides further evidence against the illicit trade narrative that tobacco companies use to oppose tobacco control policies.^13,54,57^

While the ATPS is the only UK-wide longitudinal survey aimed at assessing the response of people who smoke or who previously smoked to standardised packaging, the findings should be considered in light of limitations. The findings may not be generalisable due to the non-probability sampling design^29^ and over-representation of participants from occupational social grade ABC1, which likely reduced overall estimates of illicit tobacco in this study given that illicit tobacco use is higher among those in occupational social grade C2DE.^58^ This may explain what appears to be low rates of illicit (WSF) use across waves, and if so our findings could underestimate the scale of the problem in the UK. In addition, attrition across waves, while typical in online longitudinal tobacco studies,^59^ may have influenced the findings. The baseline sample size was also driven by practical considerations rather than power analyses. However, the comparison between W1 and W4 could not be underpowered as we found a statistically significant result in the adjusted and unadjusted models, thus a type 2 error on the overall direction of effect between W1 and W4 is not possible.

Another limitation is that we used self-reporting as a proxy measure of illicit (WSF) cigarette or RYO purchasing behaviours.^9^ Responses may have also been affected by social-desirability bias.^60^ We did not control for factors such as seasonality or other tobacco control policies, which have influenced illicit (WSF) purchasing behaviour.^9^ Although we were not able to control for other tobacco policies, the main effect of tax increases and a ban of cigarettes with characterising flavours would theoretically be to increase illicit trade. We found no evidence of an increase in illicit (WSF) tobacco use. It is possible, however, that an increase in customs and/or trading standards activity counteracted an increased demand for illicit tobacco products post-standardised packaging.

None of the questions developed by the authors to assess illicit (WSF) cigarette or RYO use or cost were pre-tested. Although the findings provide insight into illicit tobacco trade in the UK, which can be measured in several ways despite being inherently difficult to do so given its illegal nature,^47,61,62^ we cannot be sure whether participants understood the questions or knew whether the products were actually illicit (e.g. smuggled). Furthermore, some participants may have been reluctant to divulge information about clandestine practices.^35^ Nonetheless, we specifically, and comprehensively, asked about several types of potentially illicit products (e.g. no or incorrect warnings) as well as last and usual source of purchase, which increases confidence we captured those using illicit products. Despite these limitations, we can triangulate our results by comparing the results from our longitudinal analysis to Vincent et al’s^9^ repeat cross-sectional design, and with estimates from HM Revenue and Customs on tobacco tax gaps.^63^ The findings converge and suggest no increase in illicit tobacco consumption since 2016. Future research could explore the extent to which people who buy illicit tobacco products understand whether they are illicit and help improve measures of illicit purchase (e.g. types, sources).

## Conclusions

Illicit tobacco products remain an issue in the UK, as elsewhere, by helping to sustain tobacco use.^5,10-12,38^ However, we found low reported use of potentially illicit (WSF) cigarettes or RYO among adults who smoke across the four waves, with low price, availability and opportunism the key reported drivers of use. We did not find any evidence that standardised packaging in the UK was associated with an increase in illicit cigarette or RYO use. Indeed, reported illicit use was significantly lower in 2022 (five years post-standardised packaging) than in 2016. The findings help defend the decision to introduce this policy in the UK and may support the introduction of standardised packaging elsewhere.

## Supplemental Material

Supplemental Material - No Change in Illicit Tobacco Use Following the Introduction of Standardised Packaging? A Longitudinal Online Survey in the United Kingdom Supplemental Material for No Change in Illicit Tobacco Use Following the Introduction of Standardised Packaging? A Longitudinal Online Survey in the United Kingdom by Tobacco Use Insights

## Data Availability

The dataset used and analysed during the current study will be available from June 2026 from the corresponding author, CM, on reasonable request.*
