# Supplementary material for: No Change in Illicit Tobacco Use Following the Introduction of Standardised Packaging? A Longitudinal Online Survey in the United Kingdom
Source: Tob Use Insights. 2025 Dec 18;18:1179173X251405166. doi: 10.1177/1179173X251405166 (PMC12715163; doi:10.1177/1179173X251405166)
Supplement: Supplemental Material - No Change in Illicit Tobacco Use Following the Introduction of Standardised Packaging? A Longitudinal Online Survey in the United Kingdom [file sj-pdf-1-tui-10.1177_1179173X251405166.pdf]

Supplementary Table 1: Sample characteristics by survey wave

		Survey wave				
		1<br>n<br>(%)	2<br>n<br>(%)	3<br>n<br>(%)	4<br>n<br>(%)	Total<br>n<br>(%)
**Highest level of education**	High school or less	2032<br>(32.6%)	1444<br>(34.1%)	1082<br>(34.8%)	1034<br>(33.9%)	5592<br>(33.6%)
	Technical, trade school, A levels, community college	1610<br>(25.8%)	981<br>(23.2%)	701<br>(22.5%)	679<br>(22.3%)	3971<br>(23.9%)
	At least university degree	2396<br>(38.4%)	1671<br>(39.5%)	1242<br>(39.9%)	1247<br>(40.9%)	6556<br>(39.4%)
	Don't know / Prefer not to say	195<br>(3.1%)	140<br>(3.3%)	87<br>(2.8%)	87<br>(2.9%)	509<br>(3.1%)
**Gross household income**	Under £30,000	2840<br>(45.6%)	1886<br>(44.5%)	1333<br>(42.8%)	1191<br>(39.1%)	7250<br>(43.6%)
	£30,000 to £44,999	1294<br>(20.8%)	886<br>(20.9%)	656<br>(21.1%)	660<br>(21.7%)	3496<br>(21.0%)
	£45,000 and over	969<br>(15.6%)	654<br>(15.4%)	549<br>(17.6%)	627<br>(20.6%)	2799<br>(16.8%)
	Don't know / Prefer not to answer	1130<br>(18.1%)	810<br>(19.1%)	574<br>(18.4%)	569<br>(18.7%)	3083<br>(18.5%)
**Employment status**	Not employed	2505<br>(40.2%)	1683<br>(39.7%)	1248<br>(40.1%)	1265<br>(41.5%)	6701<br>(40.3%)
	Employed	3728<br>(59.8%)	2553<br>(60.3%)	1864<br>(59.9%)	1782<br>(58.5%)	9927<br>(59.7%)
**Work type**	Other or have never	832	577	350	366	2125

	worked					
		(13.4%)	(13.6%)	(11.3%)	(12.0%)	(12.8%)
	Above skilled manual	4083	2769	2123	2081	11056
		(65.5%)	(65.4%)	(68.2%)	(68.3%)	(66.5%)
	Manual	1318	890	639	600	3447
		(21.2%)	(21.0%)	(20.5%)	(19.7%)	(20.7%)
**Occupational social grade**	ABC1	3583	2381	1775	1725	9464
		(57.5%)	(56.2%)	(57.0%)	(56.6%)	(56.9%)
	C2DE	2472	1742	1292	1166	6672
		(39.7%)	(41.1%)	(41.5%)	(38.3%)	(40.1%)
	Refused / Unknown	178	113	45	156	492
		(2.9%)	(2.7%)	(1.5%)	(5.1%)	(3.0%)
**Sex**	Male	2889	2006	1519	1419	7833
		(46.4%)	(46.7%)	(47.8%)	(46.6%)	(46.8%)
	Female	3344	2287	1656	1628	8915
		(53.7%)	(53.3%)	(52.2%)	(53.4%)	(53.2%)
**Total**		6233	4293	3175	3047	16748

Supplementary Table 2: Source of last cigarette/RYO purchase

**Source**	Wave 1<br>n<br>(%)	Wave 2<br>n<br>(%)	Wave 3<br>n<br>(%)	Wave 4<br>n<br>(%)
**Licit**				
Supermarket	2442<br>(39.7%)	1575<br>(43.7%)	1124<br>(46.8%)	975<br>(50.4%)
Newsagent \ Off licence \ Corner shop	2453<br>(39.7%)	1155<br>(32.0%)	723<br>(30.1%)	586<br>(30.3%)
Duty-free shop	275<br>(4.5%)	237<br>(6.6%)	138<br>(5.8%)	112<br>(5.8%)
Petrol station	314<br>(5.1%)	171<br>(4.7%)	103<br>(4.3%)	79<br>(4.1%)
**Potentially illicit**				
Outside the UK, but NOT a duty-free shop	254<br>(4.1%)	210<br>(5.8%)	145<br>(6.0%)	52<br>(2.7%)
From friends, relatives or work colleagues	163<br>(2.6%)	91<br>(2.5%)	57<br>(2.4%)	36<br>(1.9%)
Internet	26<br>(0.4%)	16<br>(0.4%)	18<br>(0.8%)	19<br>(1.0%)
Pub (behind the bar)	11<br>(0.2%)	4<br>(0.1%)	3<br>(0.1%)	2<br>(0.1%)
**Illicit**				
Newsagent \ Off licence \ Corner shop – “cheaply, under the counter"	74	40	29	29

	(1.2%)	(1.1%)	(1.2%)	(1.5%)
Someone who sells cigarettes/RYO cheaply on street or from house/flat	39<br>(0.6%)	30<br>(0.8%)	11<br>(0.5%)	9<br>(0.5%)
Pub (someone who comes round selling cigarettes/RYO cheaply)	20<br>(0.3%)	10<br>(0.3%)	7<br>(0.3%)	6<br>(0.3%)
Someone who sells cigarettes/RYO cheaply at a market stall, car boot sale or from a mobile van	10<br>(0.2%)	5<br>(0.1%)	4<br>(0.2%)	4<br>(0.2%)
**Miscellaneous**				
Other	36<br>(0.6%)	30<br>(0.8%)	17<br>(0.7%)	13<br>(0.7%)
Have not bought	33<br>(0.5%)	23<br>(0.6%)	11<br>(0.5%)	6<br>(0.3%)
Don’t know	17<br>(0.3%)	9<br>(0.3%)	12<br>(0.5%)	6<br>(0.3%)

Supplementary Table 3: Source of usual cigarette/RYO purchase

Source	Wave 1 n (%)	Wave 2 n (%)	Wave 3 n (%)	Wave 4 n (%)
**Licit**				
Supermarket	2659 (42.9%)	1773 (49.2%)	1234 (51.3%)	1095 (56.7%)
Newsagent \ Off licence \ Corner shop	2510 (40.5%)	1186 (32.9%)	723 (30.1%)	571 (29.6%)
Duty-free shop	152 (2.5%)	123 (3.4%)	92 (3.8%)	43 (2.2%)
Petrol station	210 (3.4%)	122 (3.4%)	70 (2.9%)	48 (2.5%)
**Potentially illicit**				
Outside the UK, but NOT a duty-free shop	233 (3.8%)	161 (4.5%)	124 (5.2%)	36 (1.9%)
From friends, relatives or work colleagues	143 (2.3%)	73 (2.0%)	51 (2.1%)	26 (1.4%)
Internet	22 (0.4%)	14 (0.4%)	15 (0.6%)	22 (1.1%)
Pub (behind the bar)	10 (0.2%)	5 (0.1%)	2 (0.1%)	2 (0.1%)
**Illicit**				
Newsagent \ Off licence \ Corner	78	43	30	24

shop – “cheaply, under the counter"	(1.3%)	(1.2%)	(1.3%)	(1.2%)
Someone who sells cigarettes/RYO cheaply on street or from house/flat	39<br>(0.6%)	31<br>(0.9%)	13<br>(0.5%)	14<br>(0.7%)
Pub (someone who comes round selling cigarettes/RYO cheaply)	16<br>(0.3%)	9<br>(0.3%)	5<br>(0.2%)	2<br>(0.1%)
Someone who sells cigarettes/RYO cheaply at a market stall, car boot sale or from a mobile van	11<br>(0.2%)	6<br>(0.2%)	6<br>(0.3%)	5<br>(0.3%)
**Miscellaneous**				
Other	73<br>(1.2%)	47<br>(1.3%)	28<br>(1.2%)	22<br>(1.1%)
Don’t know	42<br>(0.7%)	13<br>(0.4%)	11<br>(0.5%)	21<br>(1.1%)

Supplementary Table 4: Complete case analysis

**Buying/receiving illicit**	W2 vs W1 OR (95% CI)	W3 vs W1 OR (95% CI)	W4 vs W1 OR (95% CI)	Over all waves (constant)
Unadjusted	1.12 (0.95-1.31)	0.86 (0.72-1.03)	0.87 (0.71-1.06)	0.13 (0.11-0.15)
Adjusted*	1.12 (0.95-1.33)	0.86 (0.71-1.03)	0.86 (0.70-1.06)	0.53 (0.31-0.89)
Effect of occupational social grade C2DE vs ABC1	1.01 (0.73-1.39)	1.04 (0.73-1.50)	**0.65 (0.43-0.97)**	0.12 (0.09-0.14)

*In the complete case analysis it becomes a fully within subjects analysis so there is no effect of adjustment for participant characteristics and unadjusted and adjusted effect estimates are identical.

Supplementary Figure 1: Predictive margins of illicit purchase by survey wave and occupational social grade

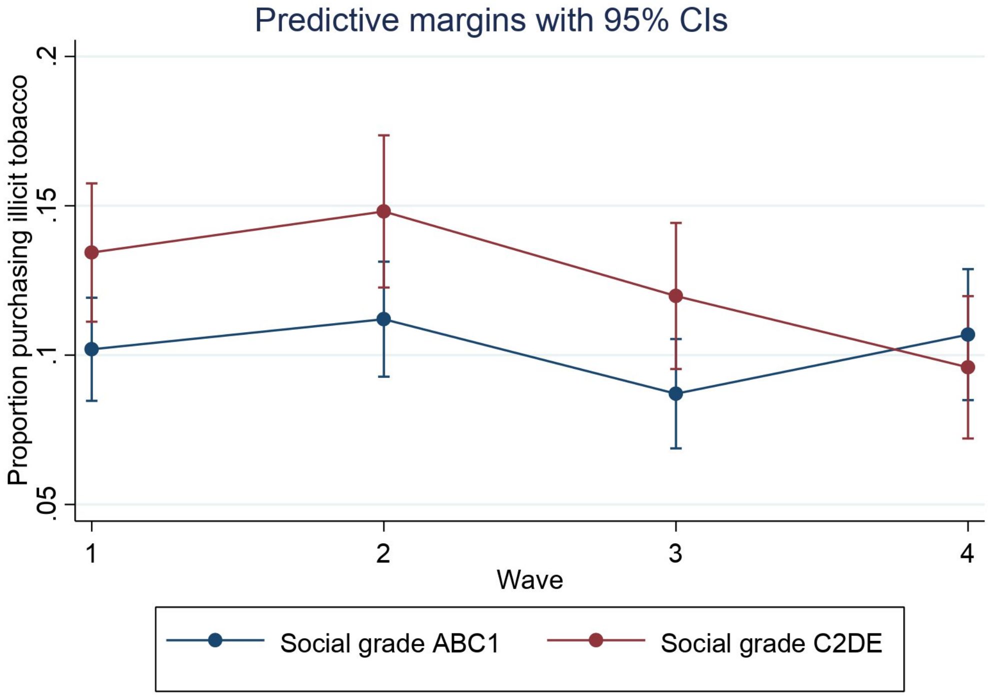

INFORMATION AND CONSENT PAGE

You are being invited to take part in a study called the 'Adult Tobacco Policy Survey', which is being run by researchers from the University of Stirling. You previously completed the survey in 2016 [*for participants that have only completed Wave 1*], 2017 [*for participants that completed Waves 1 and 2]* and 2019 [*for participants that also completed Wave 3*] (**for participants that completed only two waves, it should be '2016 and [*add in year*]'**). While you have taken part before, it is important to remind yourself of what it will involve. Please take time to read the following information.

What is the aim of the study?

We want to find out about smokers' and ex-smokers' views of tobacco policy and their current or past smoking behaviour.

What will you be asked to do?

You will be asked about tobacco policy and your current or past smoking behaviour. The survey may take up to 20 minutes to complete.

Do I have to take part?

Participation is voluntary. You can refuse to take part or exit the survey at any time, without any penalty, by closing the browser.

Are there any risks?

There are no foreseeable risks involved in participating in this survey.

What data or information will be collected?

The research team will have access to your responses to the survey, and some of the personal data you have supplied to YouGov, if you complete the survey.

What will happen to the data collected?

Your answers will be anonymous, and we will use all reasonable efforts to keep them confidential. The data will be stored in a secure location so that only members of the research team can access it. The anonymous data will be used in academic research presentations and publications but will not contain information that could potentially identify participants - you are welcome to request a copy of any publications using the contact details provided below. Data obtained as a result of the research will be retained for 10 years in secure storage. You can find out more about how your answers will be handled by the researchers by reading the separate 'Privacy Notice' (*embed link to Privacy notice*).

If you wish to withdraw your data

You have the right to withdraw your data, for any reason and without question, by the 3rd of December 2022 by writing to, emailing or phoning Crawford Moodie (details shown below).

What if you have any questions?

If you have any questions or would like to discuss the study further, either now or in the future, feel free to contact:

Crawford Moodie
Institute for Social Marketing and Health
University of Stirling
Telephone: 01786 466456
Email: c.s.moodie@stir.ac.uk

This study has been approved by the General University Ethics Panel at the University of Stirling. However, if you wish to speak to an independent advisor about the study, or have any complaints, please contact Professor Jayne Donaldson, School of Health Sciences and Sport, University of Stirling on 01786 466345. Any issues you raise will be treated in confidence and investigated, and you will be informed of the outcome.

Electronic Consent

Please select your choice below. Clicking on the "Agree" button indicates that:

- You have read and understood the above information
- You voluntarily agree to participate

Agree	☐
Disagree	☐

Screening questions	
q632a1w4	Preamble: Please note cigarettes refer to those that are factory-made (packet) and also those that are hand-rolled (rolling tobacco). Cigarettes **do not** include electronic cigarettes or vaping devices.<br><br>**In the previous survey you told us that you [*pull answer from last completed Wave*] smoke cigarettes (either factory-made or hand-rolled) every day / smoke cigarettes (either factory-made or hand-rolled) less than every day / have stopped smoking cigarettes (either factory-made or hand-rolled) / no longer smoke cigarettes (either factory-made or hand-rolled) but still smoke tobacco of some kind / did not know your current smoking status.**<br><br>**Which one of the following best describes your CURRENT smoking status?**<br>1. I smoke cigarettes (including hand-rolled) every day<br>2. I smoke cigarettes (including hand-rolled), but not every day<br>3. I do not smoke cigarettes at all, but I do smoke tobacco of some kind (e.g. Pipe, cigar or shisha)<br>4. I have stopped smoking completely in the last year (since [*add in*] 2021)<br>5. I stopped smoking completely more than a year ago (before [*add in*] 2021)<br>9. Don't know<br><br>If q632a1w4= 1 go to page Q1MM2<br>If q632a1w4= 2 go to page ADDSM<br>If q632a1w4= 3,4,5,9 go to page Q1MM2_b_2017<br>If q632a1w4= 5 AND (q632a1w2=4,5 [for those who did not complete W3] OR q632a1w3=4,5) go to STAYQUIT. Otherwise go to Q1MM2_b_2017
STAYQUIT	**BASE: (Quitters at Wave 2 who didn't complete Wave 3) or (Quitters at Wave 3) and more than one year quitters at Wave 4**<br><br>Ask if (q632a1w2=4,5 (for those who didn't complete Wave 3) OR q632a1w3=4,5) AND q632a1w4=5<br><br>You indicated that you had quit smoking when you were surveyed in [*add in*], and you just told us that you quit smoking more than year ago. Have you remained quit since you were last surveyed in [*add in*]?<br><br>1. Yes, I have remained quit since [*add in*]<br>2. No, I started smoking again since [*add in*] and then quit smoking again

ADDSM	**BASE: All who smoke but not every day (q632a1w4=2)**<br><br>**[ADDSM if q632a1w4=2] {single} Can we just confirm, how often do you **currently** smoke cigarettes (either factory-made or hand-rolled)? #SELECT ONE OPTION**<br><1> At least once a week<br><2> Less than once a week, but at least once a month<br><3> Less than once a month, but at least once in the last three months<br><4> I have not smoked cigarettes in the last three months<br><977 xor fixed> Don't know<br><br>If ADDSM=1,2,3 go to page Q1MM2<br>If ADDSM=4,977 go to page Q1MM2_b_2017

Smoking Status and other Tobacco Use	
Q1MM2	**BASE: All who currently smoke (q632a1w4=1 or ADDSM=1,2,3)**<br><br>**#ASK IF q632a1w4 = 1 or ADDSM=1,2,3**<br><br>**[Q1MM2] {multiple order=randomize} Other than cigarettes (either factory-made or hand-rolled), do you currently use any of the following tobacco products? (Please tick all that apply) #SELECT ALL THAT APPLY**<br><br><1> Cigars<br><2> Filtered cigarillos<br><3> Pipe tobacco<br><4> Shisha (also called waterpipe)<br><5> Smokeless tobacco<br><955 fixed> Other [Q1MM2_other] {open prompt="please write in"}#(please write in)<br><998 xor fixed>None of the above<br><977 xor fixed> Don't know

Q1MM2_b_2017	**BASE: Ex-smokers, other tobacco users, and don't knows to smoking status (q632a1w4=3,4,5,9) and those who have not smoked in the last three months or don't know (ADDSM=4,977)**<br><br>**#ASK IF (q632a1w4 = 3,4,5,9) OR (ADDSM=4,977)**<br><br>Do you currently use any of the following tobacco products?<br><br>**SELECT ALL THAT APPLY**<br><br>1. Cigars<br>2. Filtered cigarillos<br>3. Pipe tobacco<br>4. Shisha (also called waterpipe)<br>5. Smokeless tobacco<br>6. Other (please write in)<br>7. None of the above<br>9. Don't know

Health Knowledge	
Q111	**BASE: All**<br><br>**[Q111] {grid roworder=randomize} Below is a list of health effects that may or may not be caused by smoking cigarettes. Based on what you know or believe, does smoking cigarettes cause? #SELECT ONE OPTION FOR EACH**<br><br>**-[Q111_1] Lung cancer**<br>**-[Q111_2] Heart attacks**<br>**-[Q111_3] Diabetes**<br>**-[Q111_4] Blindness**<br>**-[Q111_5] Ageing of the skin**<br>**-[Q111_6] Damage to the teeth and gums**<br><1> Yes<br><2> No<br><977 xor fixed> Don't know

Consumption, Price, and Brands	
q632x1	**BASE: All who smoke daily (q632a1w4 = 1) or have smoked in the last three months (ADDSM = 1,2,3)**<br><br>**Ask if q632a1w4 = 1 (Smokes daily)**<br><br>**q632x1day**<br>How many cigarettes (either factory-made or hand-rolled) do you usually smoke in a typical day? We are just looking for your best estimate.<br><br>Enter number ……. (up to 99)<br><br>**Ask if ADDSM = 1 (Smokes at least weekly)**<br><br>**q632x1week**<br>How many cigarettes (either factory-made or hand-rolled) do you usually smoke in a typical week? We are just looking for your best estimate.<br><br>Enter number …….<br><br>**Ask if ADDSM = 2, 3 (Smokes at least once in the last three months)**<br><br>**q632x1month**<br>How many cigarettes (either factory-made or hand-rolled) do you usually smoke in a typical month? We are just looking for your best estimate.<br><br>Enter number …….

q632e15	**BASE: All who smoke between 1 to 300 a day/ week/ month**<br><br>**Ask, if Q632x1day OR Q632x1week OR Q632x1month = 1-300**<br><br>As you usually smoke <"XXX per day"/ "XXX per week" / "XXX per month" based on response to Q632x1>, please can you tell us how many of these you think are hand-rolled cigarettes? We are just looking for your best estimate.<br>*Enter zero if none*<br><br>Don't know = 9999<br>***YG Scripters: add another checkbox here for people who smoke none. Those who tick none or type in 0 (as there may be people who do this) are autocoded as option 2 at YGNEW. Those who type in 1+ will be shown YGNEW.***
YGNEW – This question will be hidden and auto populated.	**BASE: All who smoke both RYO or FM (q632e15=1 to 9998)**<br>**Just to confirm, in general do you tend to smoke MORE…?**<br><1>Hand rolled cigarettes<br><2>Factory-made cigarettes<br><3>Both equally
KCL_New	**BASE: All who smoke hand rolled and factory-made equally**<br><br>**#Ask if YGNEW=3**<br><br>**You indicated that you smoke both factory-made and hand-rolled cigarettes equally. Which of these did you last buy?**<br><1>Factory-made cigarettes<br><2>Hand-rolled cigarettes

ITC05	**BASE: All who currently smoke**<br>**{text if YGNew in [1,2]}**<br>**You said you smoke $ITC05_text more, therefore for this next section we want you to focus only on $ITC05_text.**<br>**{end text}**<br><br>**[ITC05] {dyngrid}Do you have a brand of...?**<br>**-[ITC05_1 if YGNew in [2] or KCL_New == 1] Factory made cigarettes that you usually smoke**<br>**-[ITC05_2 if YGNew in [1] or KCL_New == 2] Rolling tobacco that you usually smoke**<br><1> Yes<br><2> No<br><977 xor fixed> Don't know
ITC05a	**BASE: All who have a usual brand of FM they smoke**<br>**If ITC05_1=1: [ITC05a_1] And is the brand you usually smoke…** enter brand list of Factory Made brands<br><br>**AITC06b_1 if ITC05a_1 has brand] And which specific variety of cigarettes do you usually smoke?**<br><br>**BASE: All who have a usual brand of rolling tobacco they smoke**<br>**If ITC05_2=1: [ITC05a_2] And is the brand you usually smoke…** enter brand list of Rolling Tobacco brands<br><br>**[AITC06b_2 if ITC05a_2 has brand] And which specific variety of rolling tobacco do you usually smoke?**<br><br>**BASE: All who don't have a usual brand of FM they smoke**<br>**If ITC05_1=2,9:** [ITC05a_3] **You said you don't have a brand of factory-made cigarettes that you usually smoke, could you tell us the brand you are currently smoking?** Enter brand list of Factory Made Brands<br><br>**[AITC06b_3 if ITC05a_3 has brand] And which specific variety of cigarettes do you currently smoke?**<br><br>**BASE: All who don't have a usual brand of rolling tobacco they smoke**<br>**ITC05_2=2,9:** [ITC05a_4] **You said you don't have a brand of rolling tobacco that you usually smoke, could you tell us the brand you are currently smoking?** Enter brand list of Rolling Tobacco Brands<br><br>**[AITC06b_4 if ITC05a_4 has brand] And which specific variety of rolling tobacco do you currently smoke?**

ITC07	**BASE: All who usually or currently smoke FM cigarettes (ITC05_1=1 or ITC05_1=2,9)**<br><br>How many cigarettes are in your <if **ITC05_1=1**"usual pack" > <if **ITC05_1=2,9** "current pack" >? [SP]<br><br>**SELECT ONE OPTION**<br><br>1 20<br>2 23<br>3 24<br>24 I only get loose (single) cigarettes<br>25 Other (please write in) [RANGE 1-500]<br>9999 Don't Know
ITC08b	**BASE: All who usually or currently smoke RYO cigarettes (ITC05_2=1 or ITC05_2=2,9)**<br><br>What pack size of rolling tobacco <if **ITC05_2=1** "do you usually buy" > <if **ITC05_2=2,9** "are you currently using" >?<br><br>**SELECT ONE OPTION**<br><br>1 30 grams<br>2 40 grams<br>3 50 grams<br>4 100 grams<br>5 Other pack size [Enter in grams: RANGE 1-500]<br>9999 Don't Know
ITC08C_New_2017	**BASE: All who usually smoke RYO cigarettes (ITC05_2=1 or ITC05_2=2,9) and know their current pack size (ITC08b= 8 to 12)**<br><br>How many **days** does it take you to smoke this [=ITC08b] pack? (Open numeric – maximum limit 90 days)

Newcbr	**BASE: All who don't have a usual brand of cigarettes (ITC05_1=2,9)**<br>**If ITC05_1=2,9** As you don't have a usual brand of cigarettes, how do you decide on which brand to smoke?<br><br>**BASE: All who don't have a usual brand of rolling tobacco (ITC05_2=2,9)**<br>**If ITC05_2=2,9** As you don't have a usual brand of rolling tobacco, how do you decide on which brand to smoke?<br><br>**SELECT ALL THAT APPLY**<br><br>1. I buy one of the cheapest brands<br>2. I smoke the brand that my friends, family or work colleagues have<br>3. I go by the look of the pack<br>4. I only smoke other people's cigarettes<br>5. Other (please write in)<br>9. Don't Know
ITC05a1	**BASE: All who currently smoke (q632a1w4=1 or ADDSM=1,2,3)**<br><br>[ITC05a1_NEW]{single}The last time you bought tobacco - was it factory-made cigarettes or rolling tobacco?<br><1> Factory-made (packet) cigarettes<br><2> Rolling tobacco<br><977> Don't know
ASH1Pu	**BASE: All who currently smoke**<br><br>**{page pASH1Pu if ITC05a1_NEW in [1,2]}**<br>**These next questions are about the LAST time you bought $ITC05a1_NEW_text.**<br><br>**[ASH1Pu if ITC05a1_NEW in [1,2]] {single} Where did you LAST buy $ITC05a1_NEW_text for YOURSELF? #SELECT ONE OPTION**<br><1> Newsagent \ Off-licence \ Corner shop<br><2> Newsagent \ Off-license \ Corner shop – "cheaply, under the counter"<br><3> Supermarket<br><4> Petrol station<br><5> Pub (behind the bar)

<6> Duty free shop
<7> Internet
<8> From friends, relatives or work colleagues
<9> Pub (someone who comes round selling cigarettes/rolling tobacco cheaply)
<10> Someone who sells cigarettes/rolling tobacco cheaply on the street or from a house or flat
<11> Someone who sells cigarettes/rolling tobacco cheaply at a market stall, car boot sale or from a mobile van
<12> Outside the UK, but NOT a duty free shop
<13> Other [ASH1Pu_other] {open prompt="please write in"}
<14> Have not bought any cigarettes or rolling tobacco for myself in the last 6 months
<98> Don't know
{end page pASH1Pu}

If response = 8, go to ASH2Pu
Otherwise go to BR701

ASH2 Pu	**BASE: All who currently smoke and who last bought from friends, relatives or work colleagues (ASH1Pu = 8)**<br><br>**[ASH2Pu if ASH1Pu = 8] {single} Where did your friend, relative or work colleague buy the $ITC05a1_NEW_text from?**<br><br>**#SELECT ONE OPTION**<br><br><1> Newsagent \ Off-licence \ Corner shop<br><2> Newsagent \ Off-license \ Corner shop – "cheaply, under the counter"<br><3> Supermarket<br><4> Petrol station<br><5> Pub (behind the bar)<br><6> Duty free shop<br><7> Internet<br><8> Pub (someone who comes round selling cigarettes/rolling tobacco cheaply)<br><9> Someone who sells cigarettes/rolling tobacco cheaply on the street or from a house or flat<br><10> Someone who sells cigarettes/rolling tobacco cheaply at a market stall, car boot sale or from a mobile van<br><11> Outside the UK, but NOT a duty free shop<br><955 fixed> Other [ASH2Pu_other] {open prompt="please write in"}<br><977 xor fixed> Don't know

BR701	**BASE: All who bought tobacco in the last 6 months, or Don't know (ASH1Pu =1-13, OR 98)**<br><br>**[BR701_a if ITC05_1=1 and ITC05a1=1]This last time you bought cigarettes was it your usual variety(AITC06b_1)**<br><br>**[BR701_b if ITC05_2=1 and ITC05a1=2]This last time you bought rolling tobacco was it your usual variety(AITC06b_2)**<br><br>**[BR701_c if ITC05_1=2,9 and ITC05a1=1]This last time you bought cigarettes was it your current variety(AITC06b_3)**<br><br>**[BR701_d if ITC05_2=2,9 and ITC05a1=2]This last time you bought rolling tobacco was it your current variety(AITC06b_4)**<br><br>**SELECT ONE OPTION**<br>1. Yes<br>2. No<br>9. Don't know
BR731a	**BASE: All who did not buy their usual/ current brand on last purchase or who don't know (BR701_a to BR701_d=2,9)**<br><br>**If BR701_a=2,9: What specific brand of cigarettes did you last buy?**<br>**If BR701_b=2,9: What specific brand of rolling tobacco did you last buy?**<br>**If BR701_c=2,9: What specific brand of cigarettes did you last buy?**<br>**If BR701_d=2,9: What specific brand of rolling tobacco did you last buy?**<br><br>**SELECT ONE OPTION**<br><br>USE 'MASTER BRAND' LIST PROVIDED<br><br>DISPLAY APPROPRIATE CIGARETTE/ROLLING TOBACCO BRAND LIST FOR LAST PURCHASE TYPE (ITC05a1)<br><br>9997 Other (please write in and be as specific as possible)<br>9999 Don't Know

BR731b	*** Please show 4 versions of this question with the answer options showing the varieties of the brands they chose at the question above.**<br><br>**Ask if BR731a =0001-9997 (i.e. know brand or wrote in other brand)**<br><br>And which specific variety did you last buy?<br><br>**SELECT ONE OPTION**<br><br>USE 'MASTER BRAND' LIST PROVIDED<br><br>DISPLAY APPROPRIATE CIGARETTE/ROLLING TOBACCO SUB-BRAND LIST FOR BRAND CODED AT BR731a<br><br>9997 Other (please write in and be as specific as possible)<br>9999 Don't Know

PU201	**BASE: All who last bought FM from a friend or relative (ITC05a1=1 and ASH1Pu=8)**<br>**If ITC05a1=1 AND ASH1Pu = 8:** When you bought or got these cigarettes from your friend or relative, did you get them by the carton, pack, as loose (single cigarettes), or a multi-pack?<br><br>**BASE: All who last bought FM EXCLUDING from a friend or relative (ITC05a1=1 and ASH1Pu=1 to 7 or 9 to 13 or 98)**<br>**If ITC05a1=1 AND ASH1Pu = 1-7 or 9-13 or 98** Did you buy these cigarettes by the carton, pack, as loose (single cigarettes), or a multi-pack?<br><br>1 Carton (five or more packs sold together)<br>2 Pack<br>3 Loose (single cigarettes)<br>4 Multi-pack (a twin-pack or a few packs sold together)<br>9 Don't know<br><br>**If PU201 = 1, go to PU211**<br>**If PU201 = 2, go to PU311**<br>**If PU201 = 3, go to PU411**<br>**If PU201 = 4, go to PU211**<br>**If PU201=9 go to ASH3Pu**
PU211	**BASE: All who bought carton or multipacks (PU201=1 or 4)**<br><br>**Ask if PU201=1 or 4. (last purchased carton or multipack)**: Thinking about this last purchase, how many <IF PU201=1: cartons / IF PU201=4: multipacks> did you get?<br><br>**ENTER NUMBER…**<br><br>Don't Know=9999<br><br>**If** PU211 **response=1, go to** PU231**. Show** PU231 **on same screen as** PU211**.**<br>**If** PU211 **response= 2-9998, go to** PU229**. Show** PU229 **on same screen as** PU211**.**

PU229	**BASE: All who bought 2 to 9998 cartons or multipacks (PU211=2 to 9998)**<br><br>**Ask if PU211=2-9998.** We would like to find out how much you paid. Is it easier for you to say how much you paid per <IF PU201=1: carton / IF PU201=4: multipack><br>or how much you paid for all the <IF PU201=1: cartons / IF PU201=4: multipacks>?<br><br>**SELECT ONE OPTION**<br><br>1 Price per <IF PU201=1: carton / IF PU201=4: multipack><br>2 Total paid for all <IF PU201=1: cartons / IF PU201=4: multipacks><br>9 Don't know<br><br>**If PU229 response=1, go to PU231.**<br>**If PU229 response=2, go to PU241.**<br>**Otherwise, go to PU222.**
PU241	**BASE: All who want to state total price paid for carton/ multipack (PU229=2)**<br><br>**Ask if PU229=2:** What was the total price you paid for ALL [PU211] <IF PU201=1: cartons / IF PU201=4: multipacks>?<br><br>**ENTER PRICE IN POUNDS**<br>**OR**<br>**ENTER PRICE IN EUROS**<br><br>9999 Don't Know<br><br>*Please enter exact price using a decimal point in your answer. We request that you be as accurate as possible and not round up or down.*

PU231	**BASE: All who bought one carton or multipacks OR who want to give price per carton/ multipack (PU211=1 or PU229=1)**<br><br>**Ask if PU211=1 or PU229=1:** Enter the price you paid for 1 <IF PU201=1: carton / IF PU201=4: multipack>?<br><br>**ENTER PRICE IN POUNDS**<br>**OR**<br>**ENTER PRICE IN EUROS**<br><br>9999 Don't Know<br><br>*Please enter exact price using a decimal point in your answer. We request that you be as accurate as possible and not round up or down.*

PU222	**BASE: All who bought carton or multipacks (PU201=1 or 4)**<br><br>**Ask if PU201=1,4. Show PU222 and PU227 on the same screen.**<br><br>How many packs of cigarettes were in <if PU211 = 1 “THE”> / <if PU211 =2-9998 “EACH”> <IF PU201=1: carton / IF PU201=4: multipack>?<br><br>**ENTER NUMBER** …<br><br>Don't Know=9999
PU227	**BASE: ALL WHO BOUGHT CARTON OR MULTIPACKS (PU201=1 OR 4)**<br><br>**ASK IF PU201=1,4. SHOW PU222 AND PU227 ON THE SAME SCREEN.**<br><br>HOW MANY CIGARETTES WERE IN EACH PACK?<br><br>6 20<br>7 23<br>8 24<br>25 OTHER (WRITE IN) [RANGE 1-500]<br>9999 DON’T KNOW<br><br>DON’T KNOW=9999

PU2_chk	**BASE: All who bought carton or multipacks (PU201=1 or 4)**<br><br>Just to confirm:<br>You LAST bought cigarettes FOR YOURSELF by the <IF PU201=1: carton / IF PU201=4: multipack>.<br>You bought [PU211] <IF PU201=1: carton(s) / IF PU201=4: multipack(s)><br>containing [PU222] packs of cigarettes [per<IF PU201=1: carton / IF PU201=4: multipack>].<br>Each pack of cigarettes contained [PU227] cigarettes.<br>You paid [PU231 per <IF PU201=1: carton / IF PU201=4: multipack>/ PU231 for the <IF PU201=1: carton / IF PU201=4: multipack>/ PU241 for all the <IF PU201=1: cartons / IF PU201=4: multipacks> together].<br><br>Is this correct?<br>1 Yes<br>2 No<br>9 Don't know<br><br>**If PU2_chk =2, clear responses, show message below, and go back to PU201.**<br>*"You will need to answer this series of questions regarding your last purchase again. Please read each question carefully and be careful when entering your answers. Thank You."*<br>**Only ask to enter again once (one loop).*<br>**Otherwise, go to ASH3PU.**
PU311	**BASE: All who bought packs (PU201=2)**<br><br>**Ask if PU201=2:** Thinking about this last purchase, how many packs did you get?<br><br>**ENTER NUMBER…**<br><br>Don't Know=9999<br><br>**If PU311 response=1, go to PU331. Show PU331 on same screen as PU311.**<br>**If PU311 response= 2-20, go to PU329. Show PU329 on same screen as PU311.**

PU329	**BASE: All who bought 2 to 20 packs (PU311=2-20)**<br><br>**If** PU311 **response= 2-20:** We would like to find out how much you paid. Is it easier for you to say how much you paid per pack or how much you paid for all the packs?<br><br>**SELECT ONE OPTION**<br><br>1 Price per pack<br>2 Total paid for all packs<br>9 Don't know
PU341	**BASE: All who want to state total price paid for all packs (PU329=2)**<br><br>**Ask if** PU329**=2:** What was the total price you paid for ALL [PU311] packs:<br><br>**ENTER PRICE IN POUNDS**<br>**OR**<br>**ENTER PRICE IN EUROS**<br><br>9999 Don't Know<br><br>*Please enter exact price using a decimal point in your answer. We request that you be as accurate as possible and not round up or down.*

<table>
<tr><td>PU322</td><td>BASE: All who bought packs (PU201=2)

BASE: All who bought ONE pack only (PU311=1)
If PU511=1: How many cigarettes were in “THE” pack?

BASE: All who bought 2 to 20 packs (PU531=2 to 20)
If PU511=2-20: How many cigarettes were in “EACH” pack?

6 20
7 23
8 24
25 Other (write in) [RANGE 1-500]
9999 Don’t Know</td></tr>
<tr><td>PU3_chk</td><td>BASE: All who bought packs (PU201=2)

Ask if PU201=2.
Just to confirm:
You LAST bought cigarettes FOR YOURSELF by the PACK.
You bought [PU311] pack(s) containing [PU322] cigarettes [per pack].
You paid [PU331 per pack/ PU331 for the pack/ PU341 for all the packs together].

Is this correct?

1 Yes
2 No
9 Don't know

If PU3_chk=2, clear responses, show message, and go back to PU201.
“You will need to answer this series of questions regarding your last purchase again. Please read each question carefully and be careful when entering your answers. Thank You."
*Only ask to enter again once (one loop).
Otherwise, go to ASH3PU</td></tr>
</table>

PU331	**BASE: All who bought ONE pack only OR who want to state price per pack (PU311=1 or PU329=1)**<br><br>**Ask if** PU311**=1 or** PU329**=1:** Enter the price you paid for 1 pack:<br><br>**ENTER PRICE IN POUNDS**<br>**OR**<br>**ENTER PRICE IN EUROS**<br><br>9999 Don't Know<br><br>*Please enter exact price using a decimal point in your answer. We request that you be as accurate as possible and not round up or down.*
PU411	**BASE: All who bought loose (single cigarettes) (PU201=3)**<br><br>**Ask if PU201=3.**Thinking about this last purchase, how many loose cigarettes did you get?<br><br>**ENTER NUMBER…**<br><br>**Don't Know=9999**<br><br>**If PU411 response=1, go to** PU431**. Show** PU431 **on same screen as** PU411**.**<br>**If** PU411 **response= 2-200, go to** PU429**. Show** PU429 **on same screen as** PU411.

PU429	**BASE: All who bought 2 to 200 loose (single cigarettes) (PU411=2-200)**<br><br>**If** PU411 **response= 2-200:** We would like to find out how much you paid. Is it easier for you to say how much you paid per cigarette or how much you paid for all the cigarettes?<br><br>**SELECT ONE OPTION**<br><br>1 Price per cigarette<br>2 Total paid for all cigarettes<br>9 Don't know
PU441	**BASE: All who want to state total price paid for all loose cigarettes (PU429=2)**<br><br>**Ask if** PU429**=2:** What was the total price you paid for ALL [PU411] loose cigarettes:<br><br>**ENTER PRICE IN POUNDS**<br>**OR**<br>**ENTER PRICE IN EUROS**<br><br>9999 Don't Know<br><br>*Please enter exact price using a decimal point in your answer. We request that you be as accurate as possible and not round up or down.*

PU431	**BASE: All who bought ONE cigarette only OR who want to state price per cigarette (PU411=1 or PU429=1)**<br><br>**Ask if PU411=1 or PU429=1:** Enter the price you paid for each loose cigarette:<br><br>**ENTER PRICE IN POUNDS**<br>**OR**<br>**ENTER PRICE IN EUROS**<br><br>9999 Don't Know<br><br>*Please enter exact price using a decimal point in your answer. We request that you be as accurate as possible and not round up or down.*
PU4_chk	**Ask if PU201=3.**<br><br>Just to confirm:<br>You LAST bought SINGLE cigarettes FOR YOURSELF.<br>You bought [PU411] cigarette(s).<br>You paid [PU431 per cigarette/ PU431 for the cigarette/ PU441 for all the cigarettes together].<br><br>Is this correct?<br>1 Yes<br>2 No<br>9 Don't know<br><br>**If PU4_chk=2, clear responses, show message, and go back to PU201.**<br>*"You will need to answer this series of questions regarding your last purchase again. Please read each question carefully and be careful when entering your answers. Thank You."*<br>**Otherwise, go to ASH3PU**

PU511	**BASE: All who bought rolling tobacco in the last 6 months (ITC05a1=2 and ASH1Pu=1 to 13 or 98)**<br><br>Thinking about this last purchase, how many packs of rolling tobacco did you buy or get?<br>**ENTER NUMBER …**<br><br>**Don't Know=9999**<br><br>**If PU511 response=1, go to PU531. Show PU531 on same screen as PU511.**<br>**If PU511 response= 2-20, go to PU529. Show PU529 on same screen as PU511.**
PU529	**BASE: All who bought 2 to 20 packs of rolling tobacco (PU511=2 to 20)**<br><br>**If PU511 response= 2-20:** We would like to find out how much you paid. Is it easier for you to say how much you paid per pack of rolling tobacco or how much you paid for all the packs of rolling tobacco?<br><br>**SELECT ONE OPTION**<br><br>1 Price per pack of rolling tobacco<br>2 Total paid for all packs of rolling tobacco<br>9 Don't know
PU541	**BASE: All who want to state total price paid for all packs (PU529=2)**<br><br>**Ask if PU529=2:** What was the total price you paid for ALL [PU511] packs:<br><br>**ENTER PRICE IN POUNDS**<br>**OR**<br>**ENTER PRICE IN EUROS**<br><br>9999 Don't Know<br><br>*Please enter exact price using a decimal point in your answer. We request that you be as accurate as possible and not round up or down.*

PU531	**BASE: All who bought ONE pack only OR who want to state price per pack (PU511=1 or PU529=1)**<br><br>**Ask if PU511=1 or PU529=1:** Enter the price you paid for 1 rolling tobacco pack:<br><br>**ENTER PRICE IN POUNDS**<br>**OR**<br>**ENTER PRICE IN EUROS**<br><br>9999 Don't Know<br><br>*Please enter exact price using a decimal point in your answer. We request that you be as accurate as possible and not round up or down.*
PU547	**BASE: All who bought ONE pack only (PU511=1)**<br>**If PU511=1:** How many grams of rolling tobacco were in "THE" pack?<br><br>**BASE: All who bought 2 to 20 packs (PU511=2 to 20)**<br>**If PU511=2-20:** How many grams of rolling tobacco were in "EACH" pack?<br><br>1 30 grams<br>2 40 grams<br>3 50 grams<br>4 100 grams<br>5 Other pack size [Enter in grams: RANGE 1-500]<br>9999 Don't Know

PU5_chk	**BASE: All who bought rolling tobacco in the last 6 months**<br>**If ITC05a1=2 AND ASH1Pu = 1-13, OR 98**<br><br>Just to confirm:<br>You LAST bought rolling tobacco FOR YOURSELF.<br>You bought [PU511] pack(s) containing [PU547] grams [per pack].<br>You paid [PU531 per pack/ PU531 for the pack/ PU541 for all the packs together].<br><br>Is this correct?<br><br>1 Yes<br>2 No<br>9 Don't know<br><br>**If PU5_chk=2, clear responses, show message, and go back to PU511.**<br>*“You will need to answer this series of questions regarding your last purchase again. Please read each question carefully and be careful when entering your answers. Thank You."*<br>**Only ask to enter again once (one loop).*<br>**Otherwise, go to ASH3PU**

ASH3Pu	**BASE: All who have bought cigarettes/ rolling tobacco for themselves in the last 6 months (ASH1Pu=1-13 or 98)**<br><br>You told us earlier where you LAST bought =response from ITC05a1<br><br>**If YGNEW=2 or KCL_New=1: Could you tell us where you USUALLY buy cigarettes for yourself? (Please select one option)**<br>**If YGNEW=1 or KCL_New=2: Could you tell us where you USUALLY buy rolling tobacco for yourself? (Please select one option)**<br><br>**SELECT ONE OPTION**<br>1. Newsagent \ Off licence \ Corner shop<br>2. Newsagent \ Off license \ Corner shop – “cheaply, under the counter"<br>3. Supermarket<br>4. Petrol station<br>5. Pub (behind the bar)<br>6. Duty free shop<br>7. Internet<br>8. From friends, relatives or work colleagues<br>9. Pub (someone who comes round selling cigarettes/rolling tobacco cheaply)<br>10. Someone who sells cigarettes/rolling tobacco cheaply on the street or from a house or flat<br>11. Someone who sells cigarettes/rolling tobacco cheaply at a market stall, car boot sale or from a mobile van<br>12. Outside the UK, but NOT a duty free shop<br>13. Other (please write in)<br>98. Don’t know

Illicit and Price Behaviours	
CO12	**BASE: All who have bought cigarettes/ rolling tobacco for themselves in the last 6 months (ASH1Pu=1-13 or 98)**<br><br>**[CO12 if ASH1Pu in [1,2,3,4,5,6,7,8,9,10,11,12,13] or ASH2Pu in [1,2,3,4,5,6,7,8,9,10,11]]{grid roworder=randomize} In the last three months how many times, if at all, have you bought/ got packs of cigarettes or rolling tobacco _in the UK_ that…? #SELECT FROM GRID RESPONSES**<br>**-[CO12_1] Did not have health warnings, or had health warnings in a language other than English?**<br>**-[CO12_3] Might have been smuggled?**<br>**-[CO12_4] Might be fake (i.e., copies of real brands)?**<br><1> Never<br><2> Once<br><3> 2-5 times<br><4> 6-10 times<br><5> More than 10 times<br><977 xor fixed> Don't know
C012a	**BASE: All who have bought RYO or FM in the last three months that did not have health warning or had health warnings in another language (C012 (1) = 2-5)**<br><br>**[CO12a if CO12 and (CO12_1 in [2,3,4,5])] {single} For the pack(s) with no health warnings or health warnings in a language other than English, how much did you normally pay for them?**<br><1> Same price as I would pay at a shop for cigarettes or rolling tobacco ('regular price')<br><2> Less than 10% cheaper than regular price<br><3> 11-20% cheaper than regular price<br><4> 21-30% cheaper than regular price<br><5> 31-40% cheaper than regular price<br><6> 41-50% cheaper than regular price<br><7> 51-60% cheaper than regular price<br><8> 61-70% cheaper than regular price<br><9> More than 70% cheaper than regular price<br><977 xor fixed> Don't know

C012b	**BASE: All who have bought RYO or FM in the last three months that did not have health warning or had health warnings in another language (C012 (1) = 2-5)**<br><br>**[CO12b if CO12 and (CO12_1 in [2,3,4,5])] {multiple order=randomize} For the pack(s) with no health warnings or health warnings in a language other than English, why did you buy them? (Please tick all that apply)**<br><1> They were cheap<br><2> I got them while travelling outside the UK<br><3> They were available in my local area<br><4> A friend, family member or work colleague sometimes has them<br><5> The packs looked nice<br><955 fixed> Other [CO12b_other] {open prompt="please write in"} #(please write in)<br><977 xor fixed> Don't know
C012c_new	**BASE: All who have bought RYO or FM in the last three months that may have been smuggled (C012 (2) = 2-5)**<br><br>**[C012c_new if CO12 and (CO12_3 in [2,3,4,5])] {single} For the pack(s) which may have been smuggled, how much did you normally pay for them?**<br><1> Same price as I would pay at a shop for cigarettes or rolling tobacco ('regular price')<br><2> Less than 10% cheaper than regular price<br><3> 11-20% cheaper than regular price<br><4> 21-30% cheaper than regular price<br><5> 31-40% cheaper than regular price<br><6> 41-50% cheaper than regular price<br><7> 51-60% cheaper than regular price<br><8> 61-70% cheaper than regular price<br><9> More than 70% cheaper than regular price<br><977 xor fixed> Don't know

C012d	**BASE: All who have bought RYO or FM in the last three months that may have been smuggled (C012 (2) = 2-5)**<br><br>**[C012d if CO12 and (CO12_3 in [2,3,4,5])] {multiple order=randomize} For the pack(s) which may have been smuggled, why did you buy them? (Please tick all that apply)**<br><1> They were cheap<br><2> I got them while travelling outside the UK<br><3> They were available in my local area<br><4> A friend, family member or work colleague sometimes has them<br><5> The packs looked nice<br><955 fixed> Other [CO12d_other] {open prompt="please write in"}<br><977 xor fixed> Don't know
C012e_new	**BASE: All who have bought RYO or FM in the last three months that may have been fake (C012 (3) = 2-5)**<br><br>**[C012e_new if CO12 and (CO12_4 in [2,3,4,5])] {single} For the pack(s) which may have been fake, how much did you normally pay for them?**<br><1> Same price as I would pay at a shop for cigarettes or rolling tobacco ('regular price')<br><2> Less than 10% cheaper than regular price<br><3> 11-20% cheaper than regular price<br><4> 21-30% cheaper than regular price<br><5> 31-40% cheaper than regular price<br><6> 41-50% cheaper than regular price<br><7> 51-60% cheaper than regular price<br><8> 61-70% cheaper than regular price<br><9> More than 70% cheaper than regular price<br><977 xor fixed> Don't know

C012f	**BASE: All who have bought RYO or FM in the last three months that may have been fake (C012 (3) = 2-5)**<br><br>**[C012f if CO12 and (CO12_4 in [2,3,4,5])] {multiple order=randomize} For the pack(s) which may have been fake, why did you buy them? (Please tick all that apply)**<br><1> They were cheap<br><2> I got them while travelling outside the UK<br><3> They were available in my local area<br><4> A friend, family member or work colleague sometimes has them<br><5> The packs looked nice<br><955 fixed> Other [CO12f_other] {open prompt="please write in"}<br><977 xor fixed> Don’t know

Smoking dependence and cessation	
ITC20	**BASE: All who currently smoke every day q632a1w4=1**<br><br>**[ITC20 if q632a1w4=1] {single} Going back to smoking tobacco cigarettes (either factory-made or hand-rolled), how soon after you wake up do you smoke your first cigarette? #SELECT ONE OPTION**<br><1> Within 5 minutes<br><2> 6 - 30 minutes<br><3> 31 - 60 minutes<br><4> More than 60 minutes<br><977 xor fixed> Don't know
ITC20_1	**BASE: All smokers who do NOT currently smoke every day ADDSM=1,2,3**<br><br>**[ITC20_1 if ADDSM=1,2,3] {single} Going back to smoking tobacco cigarettes (either factory-made or hand-rolled), <**on days that you smoke**>, how soon after you wake up do you smoke your first cigarette? #SELECT ONE OPTION**<br><1> Within 5 minutes<br><2> 6 - 30 minutes<br><3> 31 - 60 minutes<br><4> More than 60 minutes<br><977 xor fixed> Don't know
Q632x7	**BASE: Ask all**<br><br>**[Q632x7] {single} In general, how strong have any urges to smoke been in the last 24 hours? #SELECT ONE OPTION**<br><1> I have not felt the urge to smoke in the last 24 hours<br><2> Slight<br><3> Moderate<br><4> Strong<br><5> Very strong<br><6> Extremely strong<br><977 xor fixed> Don't know

NEWQ A1	**BASE: All who currently smoke (q632a1w4=1 or ADDSM=1,2,3), and all ex-smokers who quit smoking in the last year (q632a1w4=4)**<br><br>**Ask if (q632a1w4=1, 4 or ADDSM=1,2,3)**<br><br>How many attempts, if any, have you made to quit smoking in the PAST 12 MONTHS?<br><br>**SELECT ONE OPTION**<br><br>1. No attempts<br>2. 1 attempt<br>3. 2 attempts<br>4. 3 or more attempts<br>9. Don’t Know

NEWQA1b_2017	**BASE: All smokers and all ex-smokers who stopped smoking in the last year i.e. since October/November 2021 and who have made no attempt in past 12 months, 1 or 2 quit attempts or don't know (newqa1=1,2,3,9)**<br><br>**Ask if (q632a1w4=1, 4 or ADDSM=1,2,3 AND who have made no attempt in past 12 months, 1 or 2 quit attempts or don't know (newqa1=1,2,3,9)**<br><br>**For smokers (q632a1w4=1 or ADDSM=1,2,3) and past year ex-smokers (q632a1w4= 4) do not ask this question if NEWQA1 = 4 and impute value 4 from NEWQA1 to NEWQA1b_2017 (e.g., if they made 3 or more attempts in the past 12 months, their answer should be the same since last survey because this is the upper number of quit attempts)*<br><br>You just told us that you made [=NEWQA1] in the past 12 months. We now want to find the **total** number of quit attempts you have made since the last time we surveyed you in April/May 2016 [participants that last completed Wave 1] October 2017 [participants that last completed Wave 2] May/June 2019 [participants that last completed Wave 3]<br><br>Since the last time you were surveyed (i.e. April/May 2016 [participants that last completed Wave 1] October 2017 [participants that last completed Wave 2] May/June 2019 [participants that last completed Wave 3]), how many attempts, if any [only show "if any" if NEWQA1=1 or 9], have you made to quit smoking? (Please tell us the total number of quit attempts you have made. We are just looking for your best estimate)<br><br>**SELECT ONE OPTION**<br><br>1. No attempts (do not show if NEWQA1 = 2-3)<br>2. 1 attempt (do not show if NEWQA1 = 3)<br>3. 2 attempts<br>4. 3 or more attempts (impute if NEWQA1 = 4)<br>9. Don't Know

NEWQA1c_2017	**BASE: All ex-smokers who stopped more than a year ago i.e. before October/November 2021 (q632a1w4= 5)**<br><br>**#Ask if (q632a1w4=5)** OR **(q632a1w4=5 AND** STAYQUIT=2)<br><br>Since the last time you were surveyed (i.e. April/May 2016 [participants that last completed Wave 1] October 2017 [participants that last completed Wave 2] May/June 2019 [participants that last completed Wave 3]) how many attempts, if any, have you made to quit smoking?<br><br>**SELECT ONE OPTION**<br><br>1. No attempts<br>2. 1 attempt<br>3. 2 attempts<br>4. 3 or more attempts<br>9. Don't Know
NEWQA2	**BASE: All smokers who last completed Wave 1, last completed Wave 2, or last completed Wave 3, who had never made a quit attempt or answered don't know (NEWQA2=2,977), and who have made no quit attempts since the last time they completed the survey (Wave 1, Wave 2 or Wave 3), and where (NEWQA1=1,9) and (NEWQA1b_2017=1,9)**<br><br>**#Ask if (q632a1w4=1 or ADDSM=1,2,3) AND (Wave 1 [participants that last completed Wave 1] or Wave 2 [participants that last completed Wave 2] or Wave 3 [participants that last completed Wave 3]) NEWQA2=2 or 977) AND (NEWQA1=1 or 9) AND (NEWQA1b_2017=1 or 9)**<br><br>**[NEWQA2] {single} Have you <<u>ever</u>> made an attempt to quit smoking?**<br><1> Yes<br><2> No<br><977 xor fixed> Don't know

Q207a	**BASE: Said they quit smoking in the last year but said they made no attempts in last 12 months**<br><br>**Ask if (q632a1w4=4 and newqa1=1,9)**<br><br>**You said that you stopped smoking completely in the last year (since** [*add in*] **2021) but also made no quit attempts in the past 12 months. Which of these best applies to you**?<br><br>**SELECT ONE OPTION [SP]**<br><br>1. I actually stopped smoking completely more than a year ago (before [*add in*] 2021)<br>2. Since I was successful in stopping smoking, I did not consider it to be an attempt but rather a success<br>3. I have only stopped smoking temporarily and intend to return to smoking<br>4. I have stopped smoking completely and intend to remain a non-smoker but am not ruling out the occasional puff<br>5. I stopped smoking completely without seriously attempting to do so<br>6. Other (please write in)<br>9. Don’t know

Q207b	**BASE: Said they quit smoking more than a year ago but said they made no attempts to quit since last survey**<br><br>**Ask if q632a1w4=5 and NEWQA1c_2017=1,9**<br><br>**You said that you stopped smoking completely more than a year ago (before** [*add in*] **2021) but also made no quit attempts since the last time you were surveyed (April/May 2016 [participants that last completed Wave 1] October 2017 [participants that last completed Wave 2] May/June 2019 [participants that last completed Wave 3]) when you said you were currently smoking. Which of these best applies to you**?<br><br>**SELECT ONE OPTION [SP]**<br><br>2. Since I was successful in stopping smoking, I did not consider it to be an attempt but rather a success<br>3. I have only stopped smoking temporarily and intend to return to smoking<br>4. I have stopped smoking completely and intend to remain a non-smoker but am not ruling out the occasional puff<br>5. I stopped smoking completely without attempting to do so<br>6. Other (please write in)<br>9. Don't know

newq632b8	**BASE: All smokers and ex-smokers who have made attempts to quit in the past 12 months (NEWQA1=2,3,4) OR quit in the last year but said they made no quit attempts in the last 12 months and who ticked codes 2,3,4,5 at q207a**<br><br>**Ask if (q632a1w4=1, 4 or ADDSM=1,2,3 and NEWQA1=2,3,4) OR (q632a1w4=4 and q207a=2-5))**<br><br>**[newQ632b8] {single} The next few questions relate to your <**most recent attempt to quit smoking in the past 12 months**>.<<br>> By most recent, we mean the last time you attempted to quit smoking in the past 12 months.<<br><br>>How long ago did your most recent quit attempt start? #PLEASE SELECT ONE OPTION - SP**<br><br><1> Within the last week<br><2> 1-2 weeks ago<br><3> 3-4 weeks ago<br><4> 1-3 months ago<br><5> 4-6 months ago<br><6> 7 months to 1 year ago<br><977 xor fixed> Don't know
newq632b9	**BASE WAVE 4: All smokers and ex-smokers who have made quit attempts in the past 12 months**<br><br>**#Ask if (q632a1w4=1, 4 or ADDSM=1,2,3 and NEWQA1=2,3,4) OR (q632a1w4=4 and q207a=2-5)**<br><br>**[newq632b9] {single} How long did your most recent quit attempt last before you went back to smoking? #SELECT ONE OPTION**<br><br><1> Still not smoking #(shown at Wave 2 and Wave 3 and Wave 4)<br><2> Less than 1 day<br><3> 1-6 days<br><4> 1-2 weeks #DO NOT SHOW IF newQ632b8=1]<br><5> 3-4 weeks #DO NOT SHOW IF newQ632b8=1-2]<br><6> 1-3 months #DO NOT SHOW IF newQ632b8=1-3]<br><7> 4-6 months #DO NOT SHOW IF newQ632b8=1-4]<br><8> 7 months to 1 year #DO NOT SHOW IF newQ632b8=1-5]<br><977 xor fixed> Don't know

newq632b910	**BASE Wave 4: All smokers and ex-smokers who did not make quit attempts in the past 12 months, but made quit attempts since last survey**<br><br>Ask if ((q632a1w4=1 or ADDSM=1,2,3) and (NEWQA1=1,9) and (NEWQA1b_2017=2,3,4)) OR ((q632a1w4=5) and (NEWQA1c_2017=2,3,4,9) or (Q207b=2,3,4,5))<br><br>**The next few questions relate to your <**most recent attempt to quit smoking since last survey (April/May 2016) [participants that last completed Wave 1] October 2017 [participants that last completed Wave 2] May/June 2019 [participants that last completed Wave 3])**>.<<br>> By most recent, we mean the last time you attempted to quit smoking since the last survey in (April/May 2016 [participants that last completed Wave 1] October 2017 [participants that last completed Wave 2] May/June 2019 [participants that last completed Wave 3]). How long did your most recent quit attempt last before you went back to smoking?**<br><br>**#SELECT ONE OPTION**<br><1> Still not smoking #(shown at Wave 2 and Wave 3 and Wave 4)<br><2> Less than 1 day<br><3> 1-6 days<br><4> 1-2 weeks<br><5> 3-4 weeks<br><6> 1-3 months<br><7> 4-6 months<br><8> 7 months to 1 year<br><9> More than 1 year #(show only if ((q632a1w4=1 or ADDSM=1,2,3) and (NEWQA1=1,9) and (NEWQA1b_2017=2,3,4)) OR ((q632a1w4=5) and (NEWQA1c_2017=2,3,4, 9) or (Q207b=2,3,4,5))<br><977 xor fixed> Don't know

Q632e4	**BASE: All smokers and ex-smokers who have made attempts to quit since last survey**<br><br>**Ask if NEWQA1=2-4 OR NEWQA1b_2017=2-4 OR Q207a= 1-5 OR Q207b=2-5**<br><br>**[Which, if any, of the following did you use to try to help you quit smoking during your most recent quit attempt? (Please tick all that apply) #SELECT ALL THAT APPLY**<br><br><1> Nicotine replacement product (e.g. patches/ gum/ inhaler)<br><2> Zyban (bupropion) or Champix (varenicline)<br><3> Attended a stop smoking group or one-to-one counselling/ advice/ support<br><4> Phoned the NHS stop smoking helpline<br><5> Phoned a helpline other than the NHS stop smoking helpline<br><6> Visited the NHS stop smoking website (www.nhs.uk/quit)<br><7> Visited a website other than the NHS stop smoking website<br><8> Used an application ('app') on a handheld computer (smartphone, tablet)<br><9> Electronic cigarette or vaping device<br><10> Support from family and friends<br><11> Face-to-face advice from a doctor or other health care professional (dentist, pharmacist, etc)<br><12> A tobacco product that is heated instead of burned (e.g. IQOS with Heets)<br><955 fixed> Other [Q632e4_other] {open prompt="please write in"}#(please write in)<br><966 xor fixed>Not applicable - did not use anything<br><977 xor fixed> Don't know
Q632e4 _1	**BASE: All smokers and ex-smokers who have made attempts to quit using electronic cigarette or vaping device (Q632e4=9)**<br><br>**[Q632e4_1 if Q632e4 and 9 in Q632e4] {single} What type of electronic cigarette or vaping device did you use to quit?**<br><1>A disposable e-cigarette or vaping device (non–rechargeable)<br><2>An e-cigarette or vaping device that uses replaceable pre-filled cartridges/pods (rechargeable)<br><3>An e-cigarette or vaping device with a tank that you refill with liquids (rechargeable)<br><4>A modular system that you refill with liquids (you use your own combination of separate devices: batteries, atomisers, etc.)<br><977> Don't know

QA551	**BASE: All smokers and ex-smokers who have made at least 2 attempts to quit in the last 12 months**<br><br>Ask if (q632a1w4=1,4 or ADDSM=1,2,3) AND (NEWQA1=3 to 4)<br><br>[QA551 if NEWQA1 in [2,3,4]] {single} Thinking now about ANY quit attempt in the past 12 months, what is the longest time that you have gone without smoking cigarettes in the past 12 months? #SELECT ONE OPTION<br><br><1> Less than 1 day<br><2> 1-6 days<br><3> 1-2 weeks<br><4> 3-4 weeks<br><5> 1-3 months<br><6> 4-6 months<br><7> 7 months to 1 year<br><977 xor fixed> Don't know
NewCD	**BASE: All who currently smoke (q632a1w4=1 or ADDSM=1,2,3)**<br><br>**[NewCD if q632a1w4=1 or ADDSM=1,2,3] {single} Are you currently trying to cut down on how much you smoke? #SELECT ONE OPTION**<br><1> Yes<br><2> No
NRT	**BASE: All who currently smoke (q632a1w4=1 or ADDSM=1,2,3)**<br><br>**[NRT if q632a1w4=1 or ADDSM=1,2,3] {single} How often, if at all, do you currently use nicotine replacement products (such as gums, patches, lozenges)? Please note this does not include the use of electronic cigarettes. #SELECT ONE OPTION**<br><1> Daily<br><2> Less than daily, but at least once a week<br><3> Less than weekly, but at least once a month<br><4> Less than monthly<br><5> Not at all<br><977 xor fixed> Don't know

BQ141	**BASE: All who currently smoke (q632a1w4=1 or ADDSM=1,2,3)**<br><br>**[BQ141 if q632a1w4=1 or ADDSM=1,2,3] {single} Are you planning to quit smoking? #SELECT ONE OPTION**<br><1> Within the next month<br><2> Between 1 and 6 months from now<br><3> Sometime in the future, beyond 6 months<br><4> Not planning to quit<br><977 xor fixed> Don't know
MEL1	**BASE: All who currently smoke (q632a1w4=1 or ADDSM=1,2,3)**<br><br>#Ask all<br>**[MEL1 if q632a1w4=1 or ADDSM=1,2,3] {single} In the last 30 days how many times, if any, did you stub out a cigarette before you finished it because you thought about the harms of smoking? #SELECT ONE OPTION**<br><1> Never<br><2> Once<br><3> A few times<br><4> Many times<br><977 xor fixed> Don't know
MEL2	**BASE: All who currently smoke (q632a1w4=1 or ADDSM=1,2,3)**<br><br>**[MEL2 if q632a1w4=1 or ADDSM=1,2,3] {single} In the last 30 days how many times, if any, did you stop yourself from having a cigarette when you had an urge to smoke? #SELECT ONE OPTION**<br><br><1> Never<br><2> Once<br><3> A few times<br><4> Many times<br><977 xor fixed> Don't know

	**Electronic Cigarettes**
ITC14	**BASE: All**<br><br>**We would like you to now think about electronic cigarettes, often called e-cigarettes, and similar vaping devices. These devices do not contain tobacco and produce vapour instead of smoke.**<br><br>**[ITC14] {single} Have you ever tried an electronic cigarette or vaping device? #SELECT ONE OPTION**<br><br><1> Yes<br><2> No<br><977 xor fixed> Don't know
ITC15	**BASE: All who have tried e-cigs (ITC14=1)**<br><br>**[ITC15 if ITC14 ==1] {single} How often, if at all, do you currently use an electronic cigarette or vaping device? #SELECT ONE OPTION**<br><br><1> Daily<br><2> Less than daily, but at least once a week<br><3> Less than weekly, but at least once a month<br><4> Less than monthly<br><5> Not at all<br><977 xor fixed> Don't know
ITC16	**BASE: All who use e-cigs (ITC15=1 to 4)**<br><br>**[ITC16 if ITC15 in [1,2,3,4]] {single} What type of electronic cigarette or vaping device do you currently use MOST? #SELECT ONE OPTION**<br><1> A disposable e-cigarette or vaping device (non–rechargeable)<br><2> An e-cigarette or vaping device that uses replaceable pre-filled cartridges/pods (rechargeable)<br><3> An e-cigarette or vaping device with a tank that you refill with liquids (rechargeable)<br><4> A modular system that you refill with liquids (you use your own combination of separate devices: batteries, atomisers, etc.)<br><977 xor fixed> Don't know

ITC17	**BASE: All who use e-cigs (ITC15=1 to 4)**<br><br>**Ask if ITC15 = 1-4**<br><br>**[ITC17 if ITC15 in [1,2,3,4]] {grid roworder=randomize} Why do you use electronic cigarettes or vaping devices? #SELECT ONE OPTION FOR EACH STATEMENT**<br><br>**-[ITC17_1] They may not be as bad for my health as regular cigarettes**<br>**-[ITC17_2 if q632a1w4=1 or ADDSM=1,2,3] They make it easier for me to cut down on the number of cigarettes I smoke**<br>**-[ITC17_3] So I can use them in places where smoking regular cigarettes is not allowed**<br>**-[ITC17_4 if q632a1w4=1,3 or ADDSM=1,2,3] To help me quit**<br>**-[ITC17_16 if q632a1w4=4 or 5] To help me to stay quit**<br>**-[ITC17_5] The warnings on packs of cigarettes/rolling tobacco make me think I should not be smoking**<br>**-[ITC17_6] They are cheaper than regular cigarettes**<br>**-[ITC17_7] My friends, family or work colleagues use, gifted or recommended them**<br>**-[ITC17_8] There’s no tobacco smoke**<br>**-[ITC17_9] I think they are more socially acceptable**<br>**-[ITC17_10] A health professional advised me to do so**<br>**-[ITC17_11] Because of e-cigarette advertising, promotions or sponsorship**<br>**-[ITC17_12] I don’t like the look of packs of cigarettes/rolling tobacco**<br>**-[ITC17_13] I like that I can have different flavours**<br>**-[ITC17_14] The vapour may be less harmful to people around me than cigarette smoke**<br><1> Yes<br><2> No<br><977 xor fixed> Don't know

HNB1	**BASE: All**<br><br>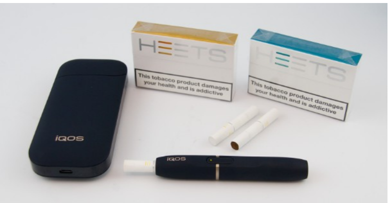<br><br>[HNB1] {single} <<img class="hnb1" src="$HNB1_image"><br>>There are new electronic products that heat tobacco instead of burning it. For this reason they are sometimes referred to as ‘heat-not-burn’ products. They use battery power to heat capsules, pods, or cigarette-like sticks that contain tobacco, and include products such as IQOS with Heets (pictured above). They are different from e-cigarettes, which do not contain tobacco. <<br><br>>Before now, had you heard about ‘heat-not-burn’ products?<br><1>Yes<br><2>No<br><977> Don’t know
HNB2	**BASE: All who are aware of heat not burn products (HNB1=1)**<br><br>Have you ever tried a heat-not-burn product?<br>1 Yes<br>2 No<br>9 Don't know
HNB3	**BASE: All who have ever tried a HNB product (HNB2=1)**<br><br>**#SELECT ALL THAT APPLY.**<br><br>Which heat-not-burn products have you ever used? (Please tick all that apply)<br>1 IQOS with Heets<br>2 Other (please write in)<br>9 Don’t know

HNB5	**BASE: All who have ever tried a HNB product (HNB2=1)**<br><br>How often, if at all, do you CURRENTLY use heat-not-burn products?<br>1 Daily<br>2 Less than daily, but at least once a week<br>3 Less than weekly, but at least once a month<br>4 Less than once a month, but occasionally<br>5 I have tried one at least several times, but no longer use at all<br>6 I have only tried once or twice<br>9 Don't know
HNB6	**BASE: All who have ever tried a HNB product (HNB2=1)**<br><br>How satisfying is using a heat-not-burn product compared to smoking ordinary cigarettes?<br>1 Much less satisfying than smoking ordinary cigarettes<br>2 Somewhat less satisfying<br>3 Equally satisfying to smoking ordinary cigarettes<br>4 Somewhat more satisfying<br>5 Much more satisfying than smoking ordinary cigarettes<br>9 Don't know
HNB7	**BASE: All who are aware of heat not burn products (HNB1=1)**<br>Compared to smoking ordinary cigarettes, how harmful do you think that using these heat-not-burn tobacco products is?<br>1 Much less harmful than smoking ordinary cigarettes<br>2 Somewhat less harmful<br>3 Equally harmful to smoking ordinary cigarettes<br>4 Somewhat more harmful<br>5 Much more harmful than smoking ordinary cigarettes<br>9 Don't know

HNB8	**BASE: All who are aware of heat not burn products (HNB1=1)**<br><br>Compared to ordinary cigarettes, how appealing do you think that heat-not-burn tobacco products are?<br>1 Much less appealing than ordinary cigarettes<br>2 Somewhat less appealing<br>3 Equally appealing to ordinary cigarettes<br>4 Somewhat more appealing<br>5 Much more appealing than ordinary cigarettes<br>9 Don't know
HNB9	**BASE: All who are aware of heat not burn products (HNB1=1)**<br><br>Compared to using **electronic** cigarettes, how harmful do you think that using heat-not-burn tobacco products is?<br>1 Much less harmful than using electronic cigarettes<br>2 Somewhat less harmful<br>3 Equally harmful to using electronic cigarettes<br>4 Somewhat more harmful<br>5 Much more harmful than using electronic cigarettes<br>9 Don't know

Warnings	
WL201	**BASE: All**<br><br>The next set of questions are about the warnings that are on packs of cigarettes and rolling tobacco.<br><br>**[WL201] {single} In the last 30 days how often, if at all, have you noticed the warning labels on packs? #SELECT ONE OPTION**<br><1> Never<br><2> Rarely<br><3> Sometimes<br><4> Often<br><5> Very often<br><977 xor fixed> Don't know
WL222	**BASE: All**<br><br>**[WL222] {single} In the last 30 days how often, if at all, have you read or looked closely at the warning labels on packs? #SELECT ONE OPTION**<br><1> Never<br><2> Rarely<br><3> Sometimes<br><4> Often<br><5> Very often<br><977 xor fixed> Don't know

WL16	**BASE: All**<br><br>**[WL16] {single} In the last 30 days how often, if at all, did you think about what the warning labels on packs are telling you? #SELECT ONE OPTION**<br><1> Never<br><2> Rarely<br><3> Sometimes<br><4> Often<br><5> Very often<br><977 xor fixed> Don't know
WL411	**BASE: All**<br><br>**[WL411] {single} To what extent, if at all, have the warning labels on packs made you think about the health risks of smoking? #SELECT ONE OPTION**<br><1> Not at all<br><2> A little<br><3> Somewhat<br><4> A lot<br><977 xor fixed> Don't know
WL221	**BASE: All**<br><br>**[WL221] {single} In the last 30 days how many times, if any, have the warning labels on packs stopped you from having a cigarette when you were about to smoke one? #SELECT ONE OPTION**<br><1> Never<br><2> Once<br><3> A few times<br><4> Many times<br><977 xor fixed> Don't know

WA11	**BASE: All who currently smoke (q632a1w4=1 or ADDSM=1,2,3)**<br><br>**PUT THESE INTO A GRID**<br><br>**[WA11 if q632a1w4=1 or ADDSM=1,2,3] {grid roworder=randomize} In the last 30 days have you done any of the following to avoid looking at the warnings on packs?**<br><br>**-[WA11_1] Avoided buying packs with particular warnings on them**<br>**-[WA11_2] Covered the warnings up to avoid looking at them**<br>**-[WA11_3] Put the pack away to avoid looking at the warning**<br>**-[WA11_4] Used a cigarette case or another pack or container to avoid looking at the warnings**<br><1> Yes<br><2> No<br><977 xor fixed> Don't know
WL421	**BASE: All who currently smoke (q632a1w4=1 or ADDSM=1,2,3)**<br><br>**[WL421 if q632a1w4=1 or ADDSM=1,2,3] {single} To what extent, if at all, do the warning labels on packs make you more likely to quit smoking? #SELECT ONE OPTION**<br><1> Not at all<br><2> A little<br><3> Somewhat<br><4> A lot<br><977 xor fixed> Don't know

WL421X	**BASE: All ex-Smokers (q632a1w4=4 or 5)**<br><br>**[WL421X IF q632a1w4=4 or 5] To what extent, if at all, do the warning labels on packs make you more likely to stay quit?**<br><br>**SELECT ONE OPTION**<br>1 Not at all<br>2 A little<br>3 Somewhat<br>4 A lot<br>9 Don't know
INSFLAP	**BASE: All who smoke RYO**<br><br>**Ask if Q632e15_1 = 1-9998 (smokes any hand rolled cigarettes)**<br><br>**[INSFLAP if q632e15_1 = 1-9998] {single} In the last 30 days how often, if at all, have you noticed warning labels on the inside of pouches of rolling tobacco? #SELECT ONE OPTION**<br><1> Never<br><2> Rarely<br><3> Sometimes<br><4> Often<br><5> Very often<br><977 xor fixed> Don't know

Anti-smoking Advertising	
ANSAD 2	**BASE: All**<br><br>**[ANSAD2] {single} In the last 30 days how often, if at all, have you seen or heard <**anti-smoking**> adverts on TV, radio, internet, billboards, posters, or in newspapers/magazines? #SELECT ONE OPTION**<br><1> Never<br><2> Rarely<br><3> Sometimes<br><4> Often<br><5> Very often<br><977 xor fixed> Don't know

Standardised Packaging	
IN619	**BASE: All who currently smoke (q632a1w4=1 or ADDSM=1,2,3)**<br><br>**If YGNEW=2 or KCL_New=1:** When you look at a pack of cigarettes, what do you usually notice first - the warning labels, or other aspects of the pack, such as branding?<br><br>**If YGNEW=1 or KCL_New=2:** When you look at a pack of rolling tobacco, what do you usually notice first - the warning labels, or other aspects of the pack, such as branding?<br><br>1 Warning labels<br>2 Other aspects of the pack, such as branding<br>9 Don't know

IN619X	**BASE: Ex-Smokers (q632a1w4=4,5) those who no longer smoke tobacco (q632a1w4=3), those who don't know their smoking status (q632a1w4=9) and those who have not smoked cigarettes in the last 3 months (ADDSM=4 or 9)**<br><br>When you look at a pack of cigarettes or rolling tobacco, what do you usually notice first - the warning labels, or other aspects of the pack, such as branding?<br><br>1 Warning labels<br>2 Other aspects of the pack, such as branding<br>9 Don't know
IN601	**BASE: All who currently smoke (q632a1w4=1 or ADDSM=1,2,3)**<br><br>**If ITC05_1=1** To what extent, if at all, do you like the look of your usual pack of cigarettes?<br>**If ITC05_2=1** To what extent, if at all, do you like the look of your usual pack of rolling tobacco?<br>**If ITC05_1=2,9** To what extent, if at all, do you like the look of your current pack of cigarettes?<br>**If ITC05_2=2,9** To what extent, if at all, do you like the look of your current pack of rolling tobacco?<br><br>**SELECT ONE OPTION**<br>1 Not at all<br>2 A little<br>3 Somewhat<br>4 Quite a lot<br>5 Very much<br>9 Don't know

PAC1	**BASE: All who currently smoke (q632a1w4=1 or ADDSM=1,2,3)**<br><br>**GRID with responses below**<br>**If ITC05_1=1** To what extent, if at all, does the look of your usual pack of cigarettes …<br>**If ITC05_2=1** To what extent, if at all, does the look of your usual pack of rolling tobacco …<br>**If ITC05_1=2,9** To what extent, if at all, does the look of your current pack of cigarettes …<br>**If ITC05_2=2,9** To what extent, if at all, does the look of your current pack of rolling tobacco …<br>1 Say something good about you to other smokers?<br>2 Make your brand stand out from other brands?<br>3 Match your style?<br><br>1 Not at all, 2 A little, 3 Somewhat, 4 Quite a lot, 5 Very much, 9 Don't know
IN90	**BASE: All who currently smoke (q632a1w4=1 or ADDSM=1,2,3)**<br><br>**[IN90_1 if ITC05_1 =1] {single}** To what extent, if at all, has the look of your usual pack of cigarettes made you think about the health risks of smoking?<br>**[IN90_2 if ITC05_2 =1] {single}** To what extent, if at all, has the look of your usual pack of rolling tobacco made you think about the health risks of smoking?<br>**[IN90_3 if ITC05_1 in [2,98]] {single}** To what extent, if at all, has the look of your current pack of cigarettes made you think about the health risks of smoking?<br>**[IN90_4 if ITC05_2 in [2,98]] {single}** To what extent, if at all, has the look of your current pack of rolling tobacco made you think about the health risks of smoking?<br><br>**SELECT ONE OPTION**<br>1 Not at all<br>2 A little<br>3 Somewhat<br>4 A lot<br>9 Don't know

IN91	**BASE: All who currently smoke (q632a1w4=1 or ADDSM=1,2,3)**<br><br>**If ITC05_1=1** In the last 30 days to what extent, if at all, has the look of your usual pack of cigarettes made you feel uncomfortable about your smoking?<br>**If ITC05_2=1** In the last 30 days to what extent, if at all, has the look of your usual pack of rolling tobacco made you feel uncomfortable about your smoking?<br>**If ITC05_1=2,9** In the last 30 days to what extent, if at all, has the look of your current pack of cigarettes made you feel uncomfortable about your smoking?<br>**If ITC05_2=2,9** In the last 30 days to what extent, if at all, has the look of your current pack of rolling tobacco made you feel uncomfortable about your smoking?<br>**SELECT ONE OPTION**<br>1 Not at all<br>2 A little<br>3 Somewhat<br>4 A lot<br>9 Don't know
NUWO	**BASE: All who currently smoke (q632a1w4=1 or ADDSM=1,2,3)**<br><br>**If ITC05_1=1** In the last 30 days to what extent, if at all, has the look of your usual pack of cigarettes made you feel worried about your smoking?<br>**If ITC05_2=1** In the last 30 days to what extent, if at all, has the look of your usual pack of rolling tobacco made you feel worried about your smoking?<br>**If ITC05_1=2,9** In the last 30 days to what extent, if at all, has the look of your current pack of cigarettes made you feel worried about your smoking?<br>**If ITC05_2=2,9** In the last 30 days to what extent, if at all, has the look of your current pack of rolling tobacco made you feel worried about your smoking?<br>**SELECT ONE OPTION**<br>1 Not at all<br>2 A little<br>3 Somewhat<br>4 A lot<br>9 Don't know

NUCF	**BASE: All who currently smoke (q632a1w4=1 or ADDSM=1,2,3)**<br><br>**If ITC05_1=1** In the last 30 days how many times, if any, has someone made a negative comment about the look of your usual cigarette pack?<br>**If ITC05_2=1** In the last 30 days how many times, if any, has someone made a negative comment about the look of your usual pack of rolling tobacco?<br>**If ITC05_1=2,9** In the last 30 days how many times, if any, has someone made a negative comment about the look of your current cigarette pack?<br>**If ITC05_2=2,9** In the last 30 days how many times, if any, has someone made a negative comment about the look of your current pack of rolling tobacco?<br>**SELECT ONE OPTION**<br>1 Never<br>2 Once<br>3 A few times<br>4 Many times<br>9 Don't know
IN92	**BASE: All who currently smoke (q632a1w4=1 or ADDSM=1,2,3)**<br><br>**If ITC05_1=1** In the last 30 days how many times, if any, has the look of your usual cigarette pack stopped you from having a cigarette when you were about to smoke one?<br>**If ITC05_2=1** In the last 30 days how many times, if any, has the look of your usual pack of rolling tobacco stopped you from having a cigarette when you were about to smoke one?<br>**If ITC05_1=2,9** In the last 30 days how many times, if any, has the look of your current cigarette pack stopped you from having a cigarette when you were about to smoke one?<br>**If ITC05_2=2,9** In the last 30 days how many times, if any, has the look of your current cigarette pack stopped you from having a cigarette when you were about to smoke one?<br>**SELECT ONE OPTION**<br>1 Never<br>2 Once<br>3 A few times<br>4 Many times<br>9 Don't know

NUSO	**BASE: All who currently smoke (q632a1w4=1 or ADDSM=1,2,3)**<br><br>**If ITC05_1=1** In the last 30 days how many times, if any, has the look of your usual cigarette pack stopped you from smoking in the company of others?<br>**If ITC05_2=1** In the last 30 days how many times, if any, has the look of your usual pack of rolling tobacco stopped you from smoking in the company of others?<br>**If ITC05_1=2,9** In the last 30 days how many times, if any, has the look of your current cigarette pack stopped you from smoking in the company of others?<br>**If ITC05_2=2,9** In the last 30 days how many times, if any, has the look of your current pack of rolling tobacco stopped you from smoking in the company of others?<br><br>**SELECT ONE OPTION**<br>1 Never<br>2 Once<br>3 A few times<br>4 Many times<br>9 Don't know
NUAV	**BASE: All who currently smoke (q632a1w4=1 or ADDSM=1,2,3)**<br><br>[NUAV if **q632a1w4=1 or ADDSM=1,2,3**] {single} In the last 30 days how many times, if any, did you deliberately cover up or conceal your pack, put it in your bag or pocket, or put your cigarettes in another container to avoid looking at it?#SELECT ONE OPTION<br><1> Never<br><2> Once<br><3> A few times<br><4> Many times<br><977 xor fixed> Don't know

IN94	**BASE: All who currently smoke (q632a1w4=1 or ADDSM=1,2,3)**<br><br>**If ITC05_1=1** To what extent, if at all, does the look of your usual cigarette pack make you more likely to quit smoking?<br>**If ITC05_2=1** To what extent, if at all, does the look of your usual pack of rolling tobacco make you more likely to quit smoking?<br>**If ITC05_1=2,9** To what extent, if at all, does the look of your current cigarette pack make you more likely to quit smoking?<br>**If ITC05_2=2,9** To what extent, if at all, does the look of your current pack of rolling tobacco make you more likely to quit smoking?<br><br>**SELECT ONE OPTION**<br><br>1 Not at all<br>2 A little<br>3 Somewhat<br>4 A lot<br>9 Don't know
IN94X	**BASE: All ex-smokers (q632a1w4=4 or 5)**<br><br>**[IN94x if q632a1w3 in ([4,5])] {single}** To what extent, if at all, does the look of packs of cigarettes or rolling tobacco help you to stay quit?<br><br><1> Not at all<br><2> A little<br><3> Somewhat<br><4> A lot<br><977 xor fixed> Don't know

H3A	**BASE: All who currently smoke (q632a1w3=1 or ADDSM=1,2,3)**<br><br>**If YGNEW=2, or KCL_New=1** Compared to the cigarettes you were smoking a year ago, how would you rate your current cigarettes in terms of …<br>**If YGNEW=1, or KCL_New=2** Compared to the rolling tobacco you were smoking a year ago, how would you rate your current rolling tobacco in terms of …<br>**GRID RESPONSES BELOW**<br><br>1 Harmfulness<br>2 Tar level<br><br>1 Higher, 2 About the same, 3 Lower, 9 Don't know, 99 Not applicable - I was not smoking a year ago

Branding	
BR11A	**BASE: All who currently smoke (q632a1w4=1 or ADDSM=1,2,3)**<br><br>**If ITC05_1=1** For the next few items we would like you to think about your usual brand of cigarettes<br>**If ITC05_2=1** For the next few items we would like you to think about your usual brand of rolling tobacco<br>**If ITC05_1=2,9** For the next few items we would like you to think about your current brand of cigarettes<br>**If ITC05_2=2,9** For the next few items we would like you to think about your current brand of rolling tobacco<br>**GRID – SEE RESPONSES BELOW**<br><br>1. Buying this brand gives me a lot of enjoyment<br>2. I find some kind of comfort in buying or having this brand<br>3. I have a lot of affection for this brand<br>4. I am drawn towards this brand<br>5. I am very attached to this brand<br>6. I like this brand<br><br>1. Strongly agree, 2. Agree, 3. Neither agree nor disagree, 4. Disagree, 5. Strongly disagree<br>9. Don't know
HAR1	**BASE: All who currently smoke (q632a1w4=1 or ADDSM=1,2,3)**<br><br>**If ITC05_1=1** Is your usual brand of cigarettes harsher or smoother on your throat than other brands?<br>**If ITC05_2=1** Is your usual brand of rolling tobacco harsher or smoother on your throat than other brands?<br>**If ITC05_1=2,9** Is your current brand of cigarettes harsher or smoother on your throat than other brands?<br>**If ITC05_2=2,9** Is your current brand of rolling tobacco harsher or smoother on your throat than other brands?<br>**SELECT ONE OPTION**<br>1 Harsher<br>2 About the same<br>3 Smoother<br>9 Don't know

FASH1	**BASE: All who currently smoke (q632a1w4=1 or ADDSM=1,2,3)**<br><br>**If ITC05_1=1** Is your usual brand of cigarettes more or less fashionable than other brands?<br>**If ITC05_2=1** Is your usual brand of rolling tobacco more or less fashionable than other brands?<br>**If ITC05_1=2,9** Is your current brand of cigarettes more or less fashionable than other brands?<br>**If ITC05_2=2,9** Is your current brand of rolling tobacco more or less fashionable than other brands?<br><br>**SELECT ONE OPTION**<br>1 More fashionable<br>2 About the same<br>3 Less fashionable<br>9 Don't know
ITCTAS	**BASE: All**<br><br>**[ITCTAS_NEWAPL_Combined]{grid} In your opinion, how much do cigarette brands differ, if at all, in how...?**<br><br>**-[ITCTAS] they taste #SELECT ONE OPTION**<br>**-[NEWAPL] appealing they are #SELECT ONE OPTION**<br><1> Not at all different<br><2> A little different<br><3> Somewhat different<br><4> Very different<br><977 xor fixed> Don't know

Capsule, Menthol, Flavours, and Harmfulness	
CAPS	**BASE: All who smoke FM (YGNEW=2 or 3)**<br><br>**Ask if YGNEW = 2 or 3**<br><br>**If ITC05_1=1** Does your usual cigarette brand have a capsule in the filter, which can be burst to change the flavour?<br>**If ITC05_1=2,9** Does your current cigarette brand have a capsule in the filter, which can be burst to change the flavour?<br>1 Yes<br>2 No<br>9 Don't know
CAPSRT	**BASE: All who smoke RYO**<br><br>**Ask if Q632e15_1 = 1-9998**<br><br>When you smoke rolling tobacco, how often, if at all, do you use filters that are flavoured (e.g. menthol) or have a capsule which can be burst to change the flavour?<br><br>1) Never<br>2) Rarely<br>3) Sometimes<br>4) Most of the time<br>5) Always<br>9) Don't know

H2	**BASE: All who currently smoke (q632a1w4=1 or ADDSM=1,2,3)**<br><br>**[H2_1 if ITC05_1==1] {single}** Now thinking about how your usual brand of cigarettes tastes...<br><br>Does your usual brand of cigarettes include any of the following flavours?<br><br>**[H2_2 if ITC05_2==1] {single}** Now thinking about how your usual brand of rolling tobacco tastes...<br><br>Does your usual brand of rolling tobacco include any of the following flavours?<br><br>**[H2_3 if ITC05_1 in [2,98]] {single}** Now thinking about how your current brand of cigarettes tastes...<br><br>Does your current brand of cigarettes include any of the following flavours?<br><br>**[H2_4 if ITC05_2 in [2,98]] {single}** Now thinking about how your current brand of rolling tobacco tastes...<br><br>Does your current brand of rolling tobacco include any of the following flavours?<br><br>**SELECT ONE OPTION**<br><br>1 Just tobacco<br>2 Tobacco and menthol<br>3 Tobacco and some other flavour (please write in)<br>9 Don't know
H211	**Ask all**<br><br>Are you aware that the sale of flavoured cigarettes and rolling tobacco, including menthol and capsule cigarettes, was banned in the UK and also across the EU in May 2020?<br><br>1 Yes<br>2 No<br>9 Don't know

H212	**Ask all**<br><br>To what extent, if at all, do you agree or disagree with the ban on the sale of flavoured cigarettes and rolling tobacco, including menthol and capsule cigarettes?<br><br>**SELECT ONE OPTION**<br><br>1 Strongly agree<br>2 Agree<br>3 Neither agree nor disagree<br>4 Disagree<br>5 Strongly disagree<br>9 Don’t know

H213	**BASE: All who used capsules or smoked menthol or flavoured cigarettes or tobacco at Wave 3**<br><br>**Ask if, at Wave 3 OR Wave 4, CAPS = 1 or CAPSRT = 2-5 or (H2_1 = 2 or H2_2 = 2 or H2_3 = 2 or H2_4 = 2) or (H2_1 = 3 or H2_2 = 3 or H2_3 = 3 or H2_4 = 3)**<br><br>Since the ban on the sale of flavoured cigarettes and rolling tobacco in May 2020 which, if any, of the following have you done?<br><br>**SELECT ALL THAT APPLY**<br><br>**Randomise except for 10, 11, 12 and 99, and keep 1-4, 5-9 together**<br><br>1) I have bought flavoured cigarettes or tobacco online<br>2) I have bought flavoured cigarettes or tobacco from another country<br>3) I have bought flavoured cigarettes or tobacco from a person or shop selling them illegally<br>4) I have bought products that could be used to flavour cigarettes or tobacco, e.g. menthol/flavour cards, menthol/flavour filters or papers, a menthol/flavour spray, loose menthol/flavour capsules<br>5) I have switched to non-flavoured cigarettes or tobacco<br>6) I have switched to other flavoured tobacco products (e.g. cigars, cigarillos, pipe tobacco)<br>7) I have switched to e-cigarettes<br>8) I have switched to 'heat-not-burn' tobacco products (electronic products that heat tobacco instead of burning it, such as IQOS)<br>9) I have switched to nicotine replacement products (such as gums, patches, lozenges)<br>10) I have quit smoking cigarettes or tobacco<br>11) Other (please write in)<br>12) None of these<br>99) Don't know

H214	**BASE: All who selected two or more responses between 1-10 at H213**<br><br>**Ask if H213 = MORE THAN ONE RESPONSE BETWEEN 1-10**<br><br>You indicated what actions you have taken since the sale of flavoured cigarettes and rolling tobacco was banned. Which of the following best applies to you now?<br><br>**SELECT ALL THAT APPLY**<br><br>*ONLY SHOW OPTIONS, FROM 1 TO 10, THAT WERE SELECTED AT H213, BUT INCLUDE OPTIONS 11 AND 12*<br><br>1) I mostly buy flavoured cigarettes or tobacco online<br>2) I mostly buy flavoured cigarettes or tobacco from another country<br>3) I mostly buy flavoured cigarettes or tobacco from a person or shop selling them illegally<br>4) I mostly buy products that could be used to flavour cigarettes or tobacco, e.g. menthol/flavour cards, menthol/flavour filters or papers, a menthol/flavour spray, loose menthol/flavour capsules<br>5) I mostly buy non-flavoured cigarettes or tobacco<br>6) I mostly buy other flavoured tobacco products (e.g. cigars, cigarillos, pipe tobacco)<br>7) I mostly buy e-cigarettes<br>8) I mostly buy 'heat-not-burn' tobacco products (electronic products that heat tobacco instead of burning it, such as IQOS)<br>9) I mostly buy nicotine replacement products (such as gums, patches, lozenges)<br>10) I have quit smoking cigarettes or tobacco<br>11) None of these<br>12) Other (please write in)<br>99) Don't know

H213a	Base: if H213=4<br><br>You indicated that you had bought products that could be used to flavour cigarettes or tobacco. How often have you used each of the following?<br><br>1 A menthol/flavour spray<br>2 Menthol/flavour cards<br>3 Menthol/flavour filters<br>4 Menthol/flavour papers<br>5 Loose menthol/flavour capsules that can be inserted into the filter<br><br>1 Never, 2 Once or twice, 3 A few times, 4 Many times, 9 Don't know
H213b	Base: if H213=4<br><br>Do you currently use this product/*these products* [if responding 2-9 for more than one option at H213a] to flavour cigarettes or tobacco?<br><br>1 A menthol/flavour spray [only show if H213a1=2-9]<br>2 Menthol/flavour cards [only show if H213a2=2-9]<br>3 Menthol/flavour filters [only show if H213a3=2-9]<br>4 Menthol/flavour papers [only show if H213a4=2-9]<br>5 Loose menthol/flavour capsules that can be inserted into the filter [only show if H213a5=2-9]<br><br>1 Yes, 2 No, 9 Don't know

H215	**BASE: All who used capsules or smoked menthol or flavoured cigarettes or tobacco at Wave 3**<br><br>**Ask if, at Wave 3, CAPS = 1 or CAPSRT = 2-5 or (H2_1 = 2 or H2_2 = 2 or H2_3 = 2 or H2_4 = 2) or (H2_1 = 3 or H2_2 = 3 or H2_3 = 3 or H2_4 = 3)**<br><br>Since the sale of flavoured cigarettes and rolling tobacco was banned, has this affected/*did this affect* [if q632a1w4=4,5] your enjoyment of smoking?<br><br>**SELECT ONE OPTION**<br><br>1 Not at all<br>2 A little<br>3 Somewhat<br>4 A lot<br>9 Don't know
H216	**BASE: All who used capsules or smoked menthol or flavoured cigarettes or tobacco at Wave 3**<br><br>**Ask if, at Wave 3, CAPS = 1 or CAPSRT = 2-5 or (H2_1 = 2 or H2_2 = 2 or H2_3 = 2 or H2_4 = 2) or (H2_1 = 3 or H2_2 = 3 or H2_3 = 3 or H2_4 = 3)**<br><br>Since the sale of flavoured cigarettes and rolling tobacco was banned, has this reduced/*did this reduce* [if q632a1w4=4,5] your desire to smoke?<br><br>**SELECT ONE OPTION**<br><br>1 Not at all<br>2 A little<br>3 Somewhat<br>4 A lot<br>9 Don't know

H3	**BASE: All who currently smoke (q632a1w4=1 or ADDSM=1,2,3)**<br><br>**If ITC05_1=1** Is your usual brand of cigarettes a little less harmful, no different, or a little more harmful, compared with other brands?<br>**If ITC05_2=1** Is your usual brand of rolling tobacco a little less harmful, no different, or a little more harmful, compared with other brands?<br>**If ITC05_1=2,9** Is your current brand of cigarettes a little less harmful, no different, or a little more harmful, compared with other brands?<br>**If ITC05_2=2,9** Is your current brand of rolling tobacco a little less harmful, no different, or a little more harmful, compared with other brands?<br><br>**SELECT ONE OPTION**<br><br>1 Little less harmful than other brands<br>2 No different<br>3 Little more harmful than other brands<br>9 Don't know
BrandT	**BASE: All who currently smoke (q632a1w4=1 or ADDSM=1,2,3)**<br><br>**If ITC05_1=1** How long have you smoked your usual brand of cigarettes for?<br>**If ITC05_2=1** How long have you smoked your usual brand of rolling tobacco for?<br>**If ITC05_1=2,9** How long have you smoked your current brand of cigarettes for?<br>**If ITC05_2=2,9** How long have you smoked your current brand of rolling tobacco for?<br><br>**SELECT ONE OPTION**<br><br><1>Less than one year<br><2>Between one and two years<br><3>Between two and three years<br><4>More than three years<br><977> Don't know

H4	**BASE: All**<br><br>**[H4_H5] {dyngrid} To what extent do you agree or disagree with the following statement:**<br>**-[H4_1] "Some brands of cigarettes contain more harmful substances than other brands"**<br>**-[H5_1] "Some brands of rolling tobacco contain less harmful ingredients than factory made cigarettes"**<br><1> Strongly agree<br><2> Agree<br><3> Neither agree nor disagree<br><4> Disagree<br><5> Strongly disagree<br><977 xor fixed> Don't know
**Psychosocial and Health Beliefs**	
HWL30	**BASE: All who currently smoke (q632a1w4=1 or ADDSM=1,2,3)**<br><br>To what extent do you agree or disagree with the following statement:<br><br>**If YGNEW=2 or KCL_New=1:** Each time I take a cigarette out I worry that I shouldn't be smoking<br>**If YGNEW=1 or KCL_New=2:** Each time I roll a cigarette I worry that I shouldn't be smoking<br><br>**SELECT ONE OPTION**<br><br>1 Strongly agree<br>2 Agree<br>3 Neither agree nor disagree<br>4 Disagree<br>5 Strongly disagree<br>9 Don’t know

Q632e38	**BASE: All who currently smoke (q632a1w4=1 or ADDSM=1,2,3)**<br><br>**[Q632e38 IF q632a1w4=1 or ADDSM=1,2,3] {grid roworder=randomize} Which of the following apply to you? Please select one option for each statement #GRID WITH RESPONSES BELOW**<br>-[Q632e38_1] I am confident I could stop smoking if I tried<br>-[Q632e38_3] I enjoy smoking<br>-[Q632e38_4] I like being a smoker<br>-[Q632e38_5] I am concerned about how much I smoke<br>-[Q632e38_6] I am addicted to smoking<br>-[Q632e38_7] I am a social smoker<br><1> Yes<br><2> No<br><977 xor fixed> Don't know

**Q632e38 _b_2017**	**BASE: All ex-smokers (q632a1w4=4 or 5)**<br><br>**Which of the following apply to you?**<br>1. I am confident I can stay quit<br>2. People I care about want me to stay quit<br>3. I enjoy not smoking<br>4. I like being a non-smoker<br>5. I still consider myself a smoker<br><br>GRID WITH RESPONSES BELOW<br>Yes=1, No=2, Don't know=9
MELEC1	**BASE: All who currently smoke (q632a1w4=1 or ADDSM=1,2,3)**<br><br>**[MELEC1 if q632a1w4=1 or ADDSM=1,2,3] {single} In the last 30 days how often, if at all, have you thought about how much you enjoy smoking? #SELECT ONE OPTION**<br><1> Never<br><2> Rarely<br><3> Sometimes<br><4> Often<br><5> Very often<br><977 xor fixed> Don't know
MELEC1 _b_2017	**BASE: All ex-smokers (q632a1w4=4 or 5)**<br><br>In the last 30 days how often, if at all, have you thought about how much you enjoyed smoking?<br><br>**SELECT ONE OPTION**<br><1> Never<br><2> Rarely<br><3> Sometimes<br><4> Often<br><5> Very often<br><977 xor fixed> Don't know

MELEC2	**BASE: All**<br><br>**[MELEC2] {single} How concerned, if at all, are you that smoking may affect or has already affected your health? #SELECT ONE OPTION**<br><1> Not at all<br><2> A little concerned<br><3> Somewhat concerned<br><4> Very concerned<br><5> Extremely concerned<br><977 xor fixed> Don't know
ROB15YSN2	**BASE: All**<br><br>**[ROB15YSN2] {single} Do you think that smoking tobacco cigarettes is? #SELECT ONE OPTION**<br><1> Very common<br><2> Common<br><3> Neither common nor uncommon<br><4> Uncommon<br><5> Very uncommon<br><977 xor fixed> Don't know
SCNORM	**BASE: All**<br><br>**[SCNORM] {single} In your opinion, do people in general approve or disapprove of people smoking tobacco cigarettes? #SELECT ONE OPTION**<br><1>Strongly approve<br><2>Approve<br><3>Neither approve nor disapprove<br><4>Disapprove<br><5>Strongly disapprove<br><977 xor fixed> Don't know

PS215	**BASE: All**<br><br>**[PS215_grid] {dyngrid} To what extent do you agree or disagree with the following statement:**<br>**-[PS215_grid_1] "People who are important to you believe that you should not smoke cigarettes."**<br><1> Strongly agree<br><2> Agree<br><3> Neither agree nor disagree<br><4> Disagree<br><5> Strongly disagree<br><977 xor fixed> Don't know

Plain Packaging, Tobacco Industry Belief, and Current Pack Being Used (current pack at Wave 2 on)	
PACUSE	**BASE: All who currently smoke (q632a1w4=1 or ADDSM=1,2,3)**<br><br>#Ask if (q632a1w4=1 or ADDSM=1,2,3)<br><br>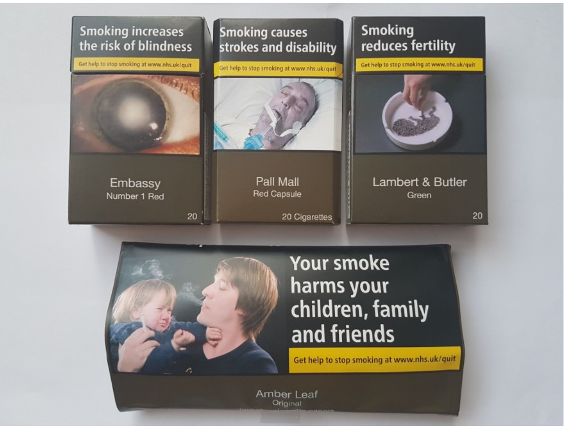<br><br><br>**Does the pack that you are currently using look like the ones shown in the image, i.e., similar colour, the brand name at the bottom, and picture warnings on the front and back?**<br>1 Yes<br>2 No<br>9 Not sure

PACUSE 2

BASE: Smokers who say No or Not sure to using standardised packs

Ask if PACUSE=2,9

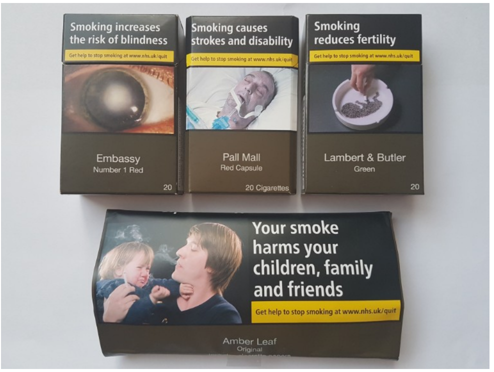

Have you previously used a pack that looks like the ones shown in the image?
1 Yes, once or twice
2 Yes, several times
3 Yes, many times
4 No
5 Not sure

FAR02

BASE: All

[FAR02_grid] {dyngrid} To what extent do you agree or disagree with the following statement:
-[FAR02_grid_1] "To help future generations, I like the idea of smoking cigarettes becoming a thing of the past."
<1> Strongly agree
<2> Agree
<3> Neither agree nor disagree
<4> Disagree
<5> Strongly disagree
<977 xor fixed> Don't know

Demographics	
DE220	**BASE: All**<br><br>**So we can better understand your answers to the previous questions, to end with there are a few questions about yourself and your household.**<br><br>**[DE220] {single} In the last 30 days, because of a shortage of money, were you unable to pay any important bills on time, such as electricity, phone or rent bills?**<br><1> Yes<br><2> No<br><977 xor fixed> Don't know<br><979 xor fixed> Prefer not to answer
profile_work_stat	**BASE: All**<br><br>**Which of these applies to you?**<br><1> Working full time (30 or more hours per week)<br><2> Working part time (8-29 hours a week)<br><3> Working part time (Less than 8 hours a week)<br><4> Full time student<br><5> Retired<br><6> Unemployed<br><7> Not working<br><8> Other<br><9> Prefer not to say

[profile_ work_typ e]	**BASE: All**<br>**Please tell us which one of the following options best describes the sort of work you do. (If you are not working now, please tell us what you did in your last job.)**<br><1> Professional or higher technical work / higher managerial - work that requires at least degree-level qualifications (e.g. doctor, accountant, schoolteacher, university lecturer, social worker, systems analyst)<br><2> Manager or Senior Administrator / intermediate managerial / professional (e.g. company director, finance manager, personnel manager, senior sales manager, senior local government officer)<br><3> Clerical/junior managerial/professional/administrator (e.g. office worker, student doctor, sales person, clerk, secretary, student teacher)<br><4> Sales or Services (e.g. commercial traveller, shop assistant, nursery nurse, care assistant, paramedic)<br><5> Foreman or Supervisor of Other Workers (e.g building site foreman, supervisor of cleaning workers)<br><6> Skilled Manual Work (e.g. plumber, electrician, fitter)<br><7> Semi-Skilled or Unskilled Manual Work (e.g. machine operator, assembler, postman, waitress, cleaner, labourer, driver, bar-worker, call centre worker)<br><8> Other<br><9> Have never worked<br><10> Prefer not to say

[profile_ethnicity]	**BASE: All**<br><br>**To which of these groups do you consider you belong?**<br><1> White British<br><2> Any other white background<br><3> White and Black Caribbean<br><4> White and Black African<br><5> White and Asian<br><6> Any other mixed background<br><7> Indian<br><8> Pakistani<br><9> Bangladeshi<br><10> Any other Asian background<br><11> Black Caribbean<br><12> Black African<br><13> Any other black background<br><14> Chinese<br><15> Other ethnic group<br><16> Prefer not to say

[profile_education_level]	**BASE: All**<br><br>**What is the highest educational or work-related qualification you have?**<br><1> No formal qualifications<br><2> Youth training certificate/skillseekers<br><3> Recognised trade apprenticeship completed<br><4> Clerical and commercial<br><5> City & Guilds certificate<br><6> City & Guilds certificate - advanced<br><7> ONC<br><8> CSE grades 2-5<br><9> CSE grade 1, GCE O level, GCSE, School Certificate<br><10> Scottish Ordinary/ Lower Certificate<br><11> GCE A level or Higher Certificate<br><12> Scottish Higher Certificate<br><13> Nursing qualification (eg SEN, SRN, SCM, RGN)<br><14> Teaching qualification (not degree)<br><15> University diploma<br><16> University or CNAA first degree (eg BA, B.Sc, B.Ed)<br><17> University or CNAA higher degree (eg M.Sc, Ph.D)<br><18> Other technical, professional or higher qualification<br><19> Don't know<br><20> Prefer not to say

[profile_gross_household]	**BASE: All**<br>**Gross HOUSEHOLD income is the combined income of all those earners in a household from all sources, including wages, salaries, or rents and before tax deductions. What is your gross household income?**<br><1> under £5,000 per year<br><2> £5,000 to £9,999 per year<br><3> £10,000 to £14,999 per year<br><4> £15,000 to £19,999 per year<br><5> £20,000 to £24,999 per year<br><6> £25,000 to £29,999 per year<br><7> £30,000 to £34,999 per year<br><8> £35,000 to £39,999 per year<br><9> £40,000 to £44,999 per year<br><10> £45,000 to £49,999 per year<br><11> £50,000 to £59,999 per year<br><12> £60,000 to £69,999 per year<br><13> £70,000 to £99,999 per year<br><14> £100,000 to £149,999 per year<br><15> £150,000 and over<br><16> Don't know<br><17> Prefer not to answer
[s6]	**BASE: All 16 to 25 year olds**<br>**{single} When you were at school did you ever receive free school meals?**<br><1>Yes, throughout my time at school<br><2>Yes some of the time<br><3> No, I never received free school meals<br><4> Don’t know<97>Not applicable<br><979> Prefer not to answer
Social grade CIE	
